# Single-photon three-qubit quantum logic using spatial light modulators

**DOI:** 10.1038/s41467-017-00580-x

**Published:** 2017-09-29

**Authors:** Kumel H. Kagalwala, Giovanni Di Giuseppe, Ayman F. Abouraddy, Bahaa E. A. Saleh

**Affiliations:** 1 CREOL, The College of Optics & Photonics, University of Central Florida, Orlando, FL 32816 USA; 2 School of Science and Technology, Physics Division, University of Camerino, Camerino, 62032 Italy

## Abstract

The information-carrying capacity of a single photon can be vastly expanded by exploiting its multiple degrees of freedom: spatial, temporal, and polarization. Although multiple qubits can be encoded per photon, to date only two-qubit single-photon quantum operations have been realized. Here, we report an experimental demonstration of three-qubit single-photon, linear, deterministic quantum gates that exploit photon polarization and the two-dimensional spatial-parity-symmetry of the transverse single-photon field. These gates are implemented using a polarization-sensitive spatial light modulator that provides a robust, non-interferometric, versatile platform for implementing controlled unitary gates. Polarization here represents the control qubit for either separable or entangling unitary operations on the two spatial-parity target qubits. Such gates help generate maximally entangled three-qubit Greenberger–Horne–Zeilinger and W states, which is confirmed by tomographical reconstruction of single-photon density matrices. This strategy provides access to a wide range of three-qubit states and operations for use in few-qubit quantum information processing protocols.

## Abstract (web summary)

Photons are essential for quantum information processing, but to date only two-qubit single-photon operations have been realized. Here the authors demonstrate experimentally a three-qubit single-photon linear deterministic quantum gate by exploiting polarization along with spatial-parity symmetry.

## Introduction

Photonic implementations of quantum logic gates^1–7^ are largely unaffected by the deleterious effects of decoherence and can potentially integrate seamlessly with existing technologies for secure quantum communication^8^. To circumnavigate challenges to realizing nonlinearity-mediated photon–photon entangling interactions^9–13^, Knill, Laflamme, and Milburn (KLM) developed a quantum computing protocol that is efficient and scalable—but probabilistic—using only single-photon sources, linear optical elements, projective measurements, and single-photon detectors^14^. In contrast, single-photon quantum logic (SPQL) aims to exploit the potentially large information-carrying capacity of a single photon to offer deterministic computation—at the expense of an exponential scale-up of resources with qubits. A quantum circuit in which a single photon encodes *n* qubits requires an interferometric network with 2^*n*^ paths^15, 16^. To curtail this exponential rise in complexity, a hybrid approach was proposed in which the degrees of freedom (DoFs) of single photons are entangled via quantum non-demolition measurements to carry out computation in smaller, spatially separated sub-systems^17^. However, this technique requires a single-photon cross-phase modulation. Few-qubit SPQL obviates the need for single-photon nonlinearities, but requires improvements in the generation and manipulation of entangled states in large-dimensional Hilbert spaces spanning all photonic DoFs—spatial, temporal, and polarization^18, 19^. Along this vein, recent efforts have focused on the spatial DoF—orbital angular momentum (OAM)^20^, spatial modes^21, 22^, or multiple paths^23–25^—in conjunction with polarization. To date, SPQL demonstrations have been limited to two qubits and include controlled-NOT (CNOT) and SWAP gates^23, 24^, implementation of the Deutsch algorithm^26^, quantum key distribution (QKD) without a shared reference frame^27^, mounting an attack on BB84 QKD^28^, and hyperentanglement-assisted Bell-state analysis^29^.

A different approach to exploiting the photon spatial DoF makes use of its spatial-parity symmetry—whether it is even or odd under inversion—regardless of the specific transverse spatial profile^30–32^. Implementing this scheme with entangled photon pairs enabled the violation of Bell’s inequality in the spatial domain exploiting Einstein–Podolsky–Rosen states^30, 33^. In this approach, the parity symmetry of a single photon can encode two qubits, one along each transverse coordinate, which provides crucial advantages. First, because only the internal transverse-parity symmetry is exploited, the need for interferometric stability required in multipath realizations is eliminated. Second, the Cartesian *x-* and *y*-coordinates of the two-dimensional (2D) transverse field are treated symmetrically, unlike the intrinsic asymmetry between the polar azimuthal and radial coordinates used in OAM experiments. Therefore, the same optical arrangement for manipulating the spatial-parity symmetry along *x* (hereafter *x*-parity for brevity) can be utilized for the *y*-parity after a rotation^34^, whereas approaches for manipulating and analyzing radial optical modes along with OAM modes are lacking (see refs. ^35–39^ for recent progress). Third, phase modulation of the single-photon wavefront—imparted by a spatial light modulator (SLM)—can rotate the qubits associated with the *x*- and *y*-parity simultaneously, and can indeed implement non-separable (entangling) rotations in their two-qubit Hilbert space^34^. These advantages point to the utility of spatial-parity-symmetry as a resource for few-qubit quantum gates^40^.

Here, we report an experimental demonstration of linear, deterministic, two- and three-qubit quantum logic gates that exploit the polarization and 2D spatial-parity-symmetry of single photons. At the center of our experiment is a polarization-selective SLM that modulates the phase of only one polarization component of the single-photon wavefront^40–42^. Because such an SLM introduces a coupling between the polarization and spatial DoFs^41^, it can implement controlled unitary gates predicated on the photon state of polarization^40^—a feature that has not received sufficient attention to date. In this conception, the photon polarization represents the “control” qubit, whereas the *x*- and *y*-parity represent the “target” qubits. The versatility of this strategy is brought to light by realizing a multiplicity of quantum gates: two-qubit CNOT and $\sqrt {{\rm{CNOT}}}$ gates, and three-qubit gates with separable or entangling controlled transformations on the target qubits. Only the phase imparted by the SLM is modified electrically—without moving parts—to select which gate is implemented. We exploit these gates to generate single-photon three-qubit maximally entangled Greenberger–Horne–Zeilinger (GHZ) and W states^43^ from initial generic, separable states. The performance of these logic gates is characterized by determining their truth tables and through quantum state tomography of the states produced when the gate is interrogated. Spatial-parity is analyzed using a balanced Mach–Zehnder interferometer (MZI) containing an optical component that flips the spatial beam profile. In lieu of traditional Dove prisms that introduce unavoidable parasitic coupling between polarization and spatial rotation^44, 45^, we exploit a custom-designed “parity prism” that is polarization neutral and thus facilitates precise projections in the parity sub-space.

Our technique is a robust approach to the photonic implementation of few-qubit quantum information processing applications. Multiple SLMs may be cascaded to realize few-qubit protocols, and potentially for implementing quantum error-correction codes^46^. The wide array of three-qubit states accessible via this technique may help improve the violations of local realistic theories in experimental tests^47^ and enhance the sensitivity of quantum metrology schemes^48^.

## Results

### Polarization and spatial parity qubits

**Fig. 1:**
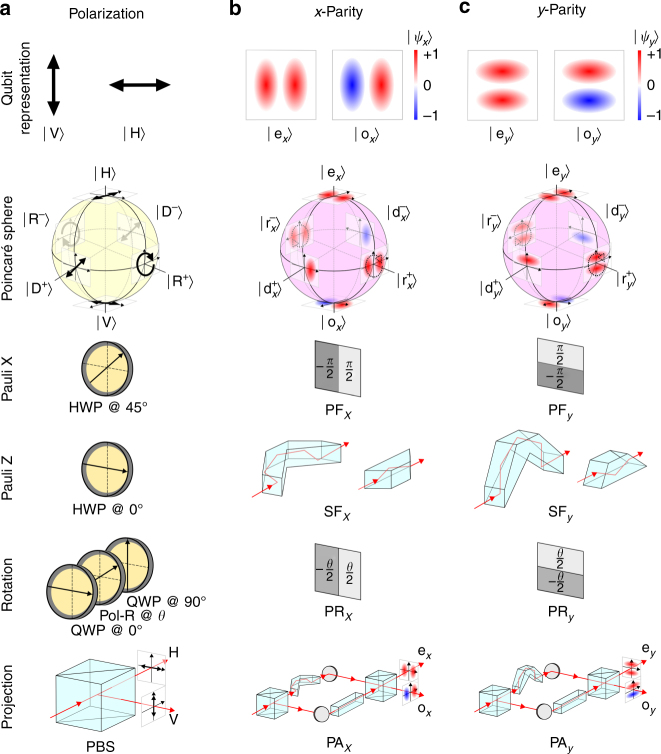
Toolbox for single-photon one-qubit operations for polarization and spatial-parity. **a** Hilbert space of polarization showing a schematic representation of the basis, the Poincaré sphere highlighting the $\left\{ {\left| {\rm{V}} \right\rangle ,\left| {\rm{H}} \right\rangle } \right\}$, $\left\{ {\left| {{{\rm{D}}^ + }} \right\rangle ,\left| {{{\rm{D}}^ - }} \right\rangle } \right\}$, and $\left\{ {\left| {{{\rm {R}}^ + }} \right\rangle ,\left| {{{\rm{R}}^ - }} \right\rangle } \right\}$ bases whose elements occupy antipodal positions; here $\left| {{{\rm{D}}^ + }} \right\rangle = \frac{1}{{\sqrt 2 }}\left( {\left| {\rm{H}} \right\rangle + \left| {\rm{V}} \right\rangle } \right)$, $\left| {{{\rm{D}}^ - }} \right\rangle = \frac{1}{{\sqrt 2 }}\left( {\left| {\rm{H}} \right\rangle - \left| {\rm{V}} \right\rangle } \right)$, $\left| {{R^ + }} \right\rangle = \frac{1}{{\sqrt 2 }}\left( {\left| {\rm{H}} \right\rangle + i\left| {\rm{V}} \right\rangle } \right)$, and $\left| {{{\rm{R}}^ - }} \right\rangle = \frac{1}{{\sqrt 2 }}\left( {\left| {\rm{H}} \right\rangle - i\left| {\rm{V}} \right\rangle } \right)$. The Pauli *X* and *Z* operators are implemented by a half-wave plate (HWP) with the fast axis oriented at 45° and 0°, respectively; a rotation operator *R*_P_(*θ*) by a sequence of a quarter-wave plate (QWP), a HWP, and a QWP, oriented at 0°, *θ*, and 0°, respectively; while a polarizing beam splitter (PBS) projects onto the $\left\{ {\left| {\rm{V}} \right\rangle ,\left| {\rm{H}} \right\rangle } \right\}$ basis. **b** Same as **a** for the *x*-parity Hilbert space. We show representative even and odd modes and the Poincaré-sphere representation of different parity bases $\left\{ {\left| {\rm{e}} \right\rangle ,\left| {\rm{o}} \right\rangle } \right\}$, $\left\{ {\left| {{{\rm{d}}^ + }} \right\rangle ,\left| {{{\rm{d}}^ - }} \right\rangle } \right\}$, and $\left\{ {\left| {{{\rm r}^ + }} \right\rangle ,\left| {{{\rm{r}}^ - }} \right\rangle } \right\}$ whose elements occupy antipodal positions; here $\left| {{{\rm{d}}^ + }} \right\rangle = \frac{1}{{\sqrt 2 }}\left( {\left| {\rm{e}} \right\rangle + \left| {\rm{o}} \right\rangle } \right)$, $\left| {{{\rm{d}}^ - }} \right\rangle = \frac{1}{{\sqrt 2 }}\left( {\left| {\rm{e}} \right\rangle - \left| {\rm{o}} \right\rangle } \right)$, $\left| {{{\rm r}^ + }} \right\rangle = \frac{1}{{\sqrt 2 }}\left( {\left| {\rm{e}} \right\rangle + i\left| {\rm{o}} \right\rangle } \right)$, and $\left| {{{\rm{r}}^ - }} \right\rangle = \frac{1}{{\sqrt 2 }}\left( {\left| {\rm{e}} \right\rangle - i\left| {\rm{o}} \right\rangle } \right)$. The parity *X*-operator (a parity flipper, PF) is implemented by a *π* phase-step along *x*; the parity *Z* (a spatial flipper, SF) by a Dove prism or a parity prism; a parity rotator (PR) by a phase-step *θ* along *x*, which rotates the state on the major circle connecting the states $\left\{ {\left| {\rm{e}} \right\rangle ,\left| {\rm{o}} \right\rangle ,\left| {{{\rm{r}}^ + }} \right\rangle ,\left| {{{\rm{r}}^ - }} \right\rangle } \right\}$; and a modified MZI acts as a parity analyzer (PA) that projects onto the {$\left| {\rm{e}} \right\rangle$,$\left| {\rm{o}} \right\rangle$} basis. **c** Same as **b** for the *y*-parity Hilbert space. All the parity-altering devices require a 90°-rotation around the propagation axis with respect to their *x*-parity counterparts

We first introduce the single-photon DoFs that will be exploited to encode quantum information. A qubit can be realized in the polarization of a single photon by associating the logical basis $\left\{ {\left| 0 \right\rangle ,\left| 1 \right\rangle } \right\}$ with the physical basis $\left\{ {\left| {\rm{V}} \right\rangle ,\left| {\rm{H}} \right\rangle } \right\}$, where $\left| {\rm{V}} \right\rangle$ and $\left| {\rm{H}} \right\rangle$ are the vertical and horizontal linear polarization components, respectively. We list relevant polarization unitary transformations as a reference for their parity counterparts. The Pauli operators $$X = \left( {\begin{array}{*{20}{c}} 0 & 1 \\ 1 & 0 \end{array}} \right)$$ and $$Z = \left( {\begin{array}{*{20}{c}}1 & 0 \\ 0 & { - 1} \end{array}} \right)$$ are each implemented by an appropriately oriented half-wave plate (HWP); a polarization rotation $${R_{\rm{P}}}\left( \theta \right) = \left( {\begin{array}{*{20}{c}} {{\rm{cos}}\frac{\theta }{2}} & {i\,{\rm{sin}}\frac{\theta }{2}} \\ {i\,{\rm{sin}}\frac{\theta }{2}} & {{\rm{cos}}\frac{\theta }{2}} \end{array}} \right)$$ by a sequence of wave plates; and projections by a polarizing beam splitter (PBS) (Fig. 1a).

A corresponding Hilbert space may be constructed for *x*-parity^30–33^ spanned by the basis $\left\{ {\left| {\rm{e}} \right\rangle ,\left| {\rm{o}} \right\rangle } \right\}$, where $\left| {\rm{e}} \right\rangle$ and $\left| {\rm{o}} \right\rangle$ correspond to even- and odd-components of the photon field distribution along *x*, respectively, and are depicted as antipodal points on a parity-Poincaré sphere (Fig. 1b). Parity is a particularly convenient embodiment of a qubit since it can be readily manipulated via simple linear optical components^30, 32^. The parity *X*-operator is implemented by a phase plate that imparts a *π* phase-step along *x*, which is thus a parity flipper: $\left| {\rm{e}} \right\rangle$ → *i* $\left| {\rm{o}} \right\rangle$ and $\left| {\rm{o}} \right\rangle$ → *i* $\left| {\rm{e}} \right\rangle$. This device multiplies the waveform by a phase factor ${e^{i\frac{\pi }{2}{\rm{sgn}}(x)}}$, where sgn(*x*) = 1 when *x* ≥ 0, and sgn(*x*) = −1 otherwise. The parity *Z*-operator is a spatial flipper *ψ*(*x*) → *ψ*(−*x*), which can be realized by a mirror, a Dove prism, or a parity prism (introduced below), resulting in the transformation: $\left| {\rm{e}} \right\rangle$ → $\left| {\rm{e}} \right\rangle$ and $\left| {\rm{o}} \right\rangle$ → −$\left| {\rm{o}} \right\rangle$. An *x*-parity rotator *R*(*θ*) that rotates parity by an angle *θ* around a major circle on the Poincaré sphere is implemented by a phase plate introducing a phase-step *θ* along *x*, an operation that multiplies the waveform by a phase factor ${e^{i\frac{\theta }{2}{\rm{sgn}}(x)}}$. A parity analyzer, realized by a MZI containing a spatial flipper in one arm, projects onto the parity basis $\left\{ {\left| {\rm{e}} \right\rangle ,\left| {\rm{o}} \right\rangle } \right\}$ (Fig. 1b). The spatial flip is performed using a “parity prism” in lieu of the traditional Dove prism. By virtue of input and output facets that are normal to the incident beam, the parity prism introduces crucial advantages for our measurements. In contradistinction to a Dove prism, the parity prism is free of polarization-dependent losses and of the parasitic coupling between polarization and spatial rotation^44, 45^. All these *x*-parity operations may be appropriated for the *y*-parity qubit by rotating the components in physical space by 90° around the propagation axis^34^ (Fig. 1c). Note that the parity prism can be rotated with high beam-pointing stability (Supplementary Note 1). The qubits encoded in the *x*- and *y*-parity can be manipulated independently by cascading optical components that impact only one transverse coordinate.

### Spatial light modulator as a two-qubit controlled-unitary gate

At the heart of our strategy for constructing deterministic SPQL is utilizing the polarization-selectivity of phase-only liquid-crystal-based SLMs^49^. Such devices impart a spatially varying phase factor *e* ^*iφ*(*x*, *y*)^ to only one polarization component of an impinging vector optical field (assumed $\left| {\rm{H}} \right\rangle$ throughout), while the orthogonal $\left| {\rm{V}} \right\rangle$ polarization component remains invariant. A coupling between the polarization and spatial DoFs is thus introduced^41, 42^, thereby entangling the associated logical qubits^40^. The two-qubit four-dimensional Hilbert space associated with polarization and *x*-parity is spanned by the hybrid basis $\left\{ {\left| {\rm{V}} \right\rangle ,\left| {\rm{H}} \right\rangle } \right\} \otimes \left\{ {\left| {\rm{e}} \right\rangle ,\left| {\rm{o}} \right\rangle } \right\} = \left\{ {\left| {{\rm{Ve}}} \right\rangle ,\left| {{\rm{Vo}}} \right\rangle ,\left| {{\rm{He}}} \right\rangle ,\left| {{\rm{Ho}}} \right\rangle } \right\}$, in correspondence with the logical basis $\left\{ {\left| {00} \right\rangle ,\left| {01} \right\rangle ,\left| {10} \right\rangle ,\left| {11} \right\rangle } \right\}$.

**Fig. 2:**
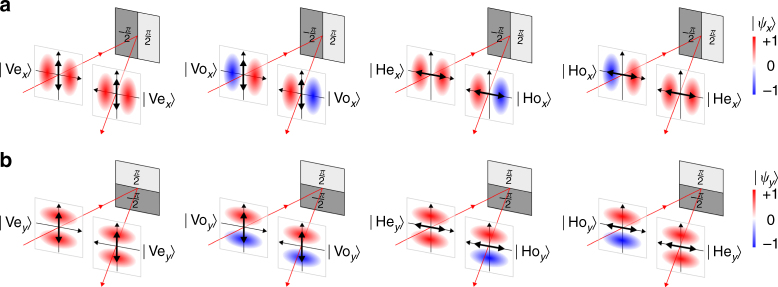
Impact of a polarization-sensitive phase-only SLM on polarized single-photons. **a** Impact of a polarization-sensitive phase-only SLM on *x*-parity. The SLM imparts a phase step *π* along *x*. When the polarization is horizontal $\left| {\rm{H}} \right\rangle$, the SLM phase modulation results in a flip in the *x*-parity: $\left| {{{\rm{e}}_x}} \right\rangle$ → *i* $\left| {{{\rm{o}}_x}} \right\rangle$ and $\left| {{{\rm{o}}_x}} \right\rangle$ → *i* $\left| {{{\rm{e}}_x}} \right\rangle$. When the polarization is vertical $\left| {\rm{V}} \right\rangle$, the *x*-parity is unchanged, regardless of the SLM phase: $\left| {{{\rm{e}}_x}} \right\rangle$ → $\left| {{{\rm{e}}_x}} \right\rangle$ and $\left| {{{\rm{o}}_x}} \right\rangle$ → $\left| {{{\rm{o}}_x}} \right\rangle$. **b** Same as **a** applied to *y*-parity. The SLMs in **a** and **b** can thus be viewed as two-qubit quantum logic gates where polarization is the control qubit and *x*- or *y*-parity is the target qubit

**Fig. 3:**
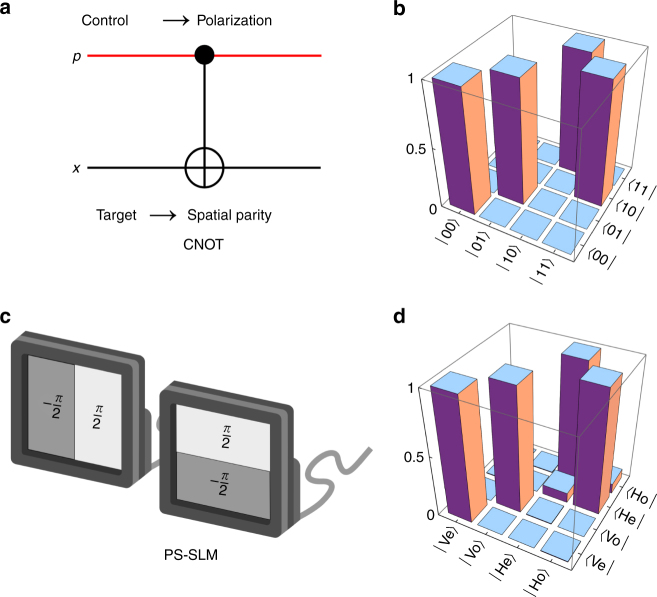
A polarization-sensitive spatial light modulator as a single-photon CNOT gate. **a** Quantum circuit of a CNOT gate and **b** the corresponding operator represented in the logical basis ${\left\{ {\left| 0 \right\rangle ,\left| 1 \right\rangle } \right\}_{{\rm{control}}}} \otimes {\left\{ {\left| 0 \right\rangle ,\left| 1 \right\rangle } \right\}_{{\rm{target}}}}$. **c** Physical realization of a CNOT gate in polarization-parity space with a spatial light modulator (SLM). The SLM imparts a phase step *π* between the two halves of the plane along either *x* or *y* and thus acts as a parity flipper when the control qubit (polarization) is on. **d** The corresponding operator represented in the $\left\{ {\left| {\rm{V}} \right\rangle ,\left| {\rm{H}} \right\rangle } \right\}$_control_ $\otimes {\left\{ {\left| {\rm{e}} \right\rangle ,\left| {\rm{o}} \right\rangle } \right\}_{{\rm{target}}}}$ basis

We illustrate the impact of an SLM imparting a phase-step *π* along *x* on the four basis states in Fig. 2a. When the polarization is $\left| {\rm{V}} \right\rangle$, the parity is invariant: $\left| {{\rm{Ve}}} \right\rangle$ → $\left| {{\rm{Ve}}} \right\rangle$ and $\left| {{\rm{Vo}}} \right\rangle$ → $\left| {{\rm{Vo}}} \right\rangle$; when the polarization is $\left| {\rm{H}} \right\rangle$, the parity is flipped: $\left| {{\rm{He}}} \right\rangle$ → *i* $\left| {{\rm{Ho}}} \right\rangle$ and $\left| {{\rm{Ho}}} \right\rangle$ → *i* $\left| {{\rm{He}}} \right\rangle$. This action is consistent with a CNOT gate with polarization and *x*-parity corresponding to the control and target qubits, respectively. Similarly, a CNOT gate with *y*-parity playing the role of the target qubit is realized when a phase-step *π* is imparted by the SLM along *y* rather than *x* (Fig. 2b). In Fig. 3 we illustrate the correspondence between the ideal truth table of a CNOT gate (Fig. 3a, b) and the measured truth table produced by the SLM implementation (Fig. 3c, d).

More generally, implementing a phase-step *θ* along *x* by an SLM rotates the parity of only the $\left| {\rm{H}} \right\rangle$ polarization. The unitary operator associated with the SLM action is represented by the matrix

1 $${U_2}(\theta ) = \left( {\begin{array}{*{20}{c}} 1 & 0 & 0 & 0 \\ 0 & 1 & 0 & 0 \\ 0 & 0 & {{\rm{cos}}\frac{\theta }{2}} & {i\,{\rm{sin}}\frac{\theta }{2}} \\ 0 & 0 & {i\,{\rm{sin}}\frac{\theta }{2}} & {{\rm{cos}}\frac{\theta }{2}} \end{array}} \right),$$

corresponding to a single-parameter controlled-unitary gate, where polarization and parity are the control and target qubits, respectively; the subscript in *U*_2_(*θ*) refers to the number of qubits involved in the gate operation. This optical realization of a two-qubit quantum gate has several salutary features. The SLM is a non-interferometric device, making the gate stable and less prone to decoherence and noise. Moreover, the phase *θ* may be varied in real-time electronically with no moving parts, thus enabling access to a continuous family of two-qubit gates in a single robust device. For instance, by setting *θ* = *π* we obtain a CNOT gate, while $\theta = \frac{\pi }{2}$ results in a $\sqrt {{\rm{CNOT}}}$ gate. Such a gate is an intermediate between the identity and CNOT, such that applying the gate twice in succession produces a CNOT gate, $\sqrt {{\rm{CNOT}}} \cdot \sqrt {{\rm{CNOT}}} = {\rm{CNOT}}$. Furthermore, a single SLM enables the manipulation of the *x*- and *y*-parity Hilbert spaces either independently or jointly^34, 40^, therefore providing a versatile platform for constructing three-qubit gates, as we demonstrate below.

### Experimental demonstration of two-qubit SPQL

**Fig. 4:**
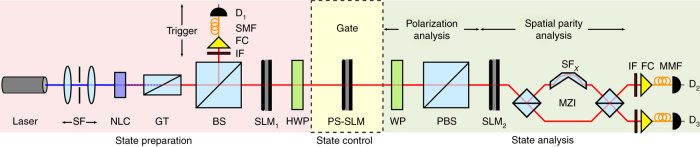
Schematic of experimental setup. SF: spatial filter, NLC: nonlinear crystal, GT: Glan–Thomson polarizer, BS: beam splitter, SLM: spatial light modulator, HWP: half-wave plate, WP: wave plate (either half-wave or quarter-wave according to the measurement basis), PBS: polarizing beam splitter, SF_*x*_: spatial flipper along *x*, MZI: Mach–Zehnder interferometer, IF: interference filter, FC: fiber coupler, MMF: multi-mode fiber, SMF: single-mode fiber, D_1_ and D_2_: single-photon-sensitive detectors. See Supplementary Note 2 for a more detailed setup layout. The spatial flipper—implemented by a parity prism—in the MZI is varied according to the measurement basis (Supplementary Note 3). All measurements are carried out by recording the detection coincidences between D_1_ and D_2_

We have verified the operation of a variety of two-qubit SPQL implemented with a SLM using the setup shown schematically in Fig. 4. Utilizing an entangled two-photon source, we project one photon onto a single spatial mode to herald the arrival of a one-photon state at the SLM-based quantum gate, which is followed by two-qubit quantum state tomography measurements on the polarization-parity space. It can be shown that type-I spontaneous parametric down-conversion (SPDC) produced from a nonlinear crystal illuminated with a strong $\left| {\rm{H}} \right\rangle$-polarized laser whose spatial profile is separable in the *x* and *y* coordinates and has even spatial parity is given by $\left| {\Psi} \right\rangle \propto \left| {{{\rm{V}}_1}{{\rm{V}}_2}} \right\rangle \otimes {\left\{ {\left| {{{\rm{e}}_1}{{\rm{e}}_2}} \right\rangle + \left| {{{\rm{o}}_1}{{\rm{o}}_2}} \right\rangle } \right\}_x}$, where the subscripts 1 and 2 identify the signal and idler photons, respectively, and the state of *y*-parity of the two photons has been traced out^30–33^. By projecting the idler photon onto the $\left| {{{\rm{e}}_{\rm{2}}}} \right\rangle$ mode via spatial filtering, a single-photon in a generic separable state in polarization-parity space $\left| {{\Psi _{\rm{i}}}} \right\rangle$ = $\left| {{\rm{Ve}}} \right\rangle$ is heralded, corresponding to logical $\left| {00} \right\rangle$ basis (Methods).

**Fig. 5:**
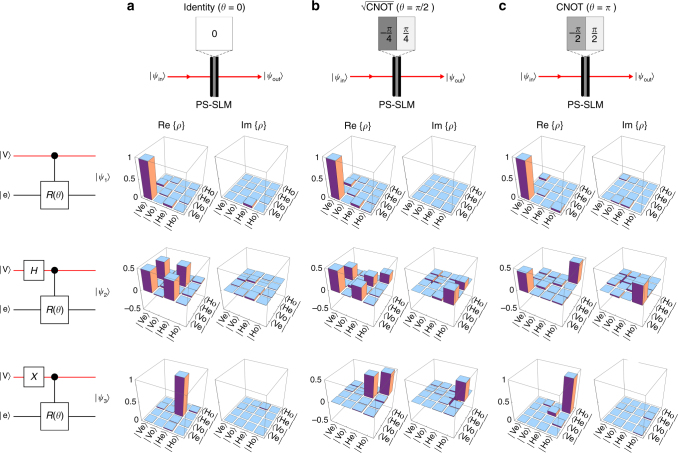
Two-qubit single-photon quantum logic gates. Characterization of three two-qubit quantum gates on the space of polarization and *x*-parity: **a** the identity gate; **b** the $\sqrt {{\rm{CNOT}}}$ gate; and **c** the CNOT gate. For each gate, we show the phase implemented on the PS-SLM (Fig. 4), and the real and imaginary parts of the density matrix Re{*ρ*} and Im{*ρ*}, respectively, reconstructed from quantum-state-tomography measurements at the quantum gate output for three different input states. The SLM implements a phase-step *θ* along *x*, **a** *θ* = 0, **b** *θ* = $\frac{\pi }{2}$, and **c** *θ* = *π* (*top row*) to create the desired gates. The three input states $\left| {{\Psi _{{\rm{in}}}}} \right\rangle$ are (ordered in the rows from *top* to *bottom*): (1) the initial separable generic state $\left| {{\rm{Ve}}} \right\rangle$ produced by the heralded source in Fig. 4; (2) $\frac{1}{{\sqrt 2 }}\left\{ {\left| {\rm{H}} \right\rangle + \left| {\rm{V}} \right\rangle } \right\} \otimes \left| {\rm{e}} \right\rangle$ obtained by rotating the polarization 45° (corresponding to the Hadamard gate *H*, a half-wave plate rotated 22.5°); and (3) $\left| {{\rm{He}}} \right\rangle$ obtained by rotating the polarization $\left| {\rm{V}} \right\rangle$ → $\left| {\rm{H}} \right\rangle$ (corresponding to the Pauli-*X* operator, a half-wave plate rotated 45°). On the leftmost column we show schematics of the combined system for initial state preparation and quantum gate

The quantum gate itself consists of a single linear optical component: the polarization-selective SLM (PS-SLM in Fig. 4). By implementing a phase-step *θ* on the SLM, we produce a controlled-unitary gate that rotates the state of *x*-parity conditioned on the polarization state (Eq. (1)). We test three settings that result in distinct two-qubit quantum gates: *θ* = 0, *θ* = $\frac{\pi }{2}$, and *θ* = *π*, corresponding to the identity gate, a $\sqrt {{\rm{CNOT}}}$ gate, and a CNOT gate, respectively. We interrogate these gates using three logical states: $\left| {00} \right\rangle$ prepared by our heralded single-photon source, corresponding to the state $\left| {{\rm{Ve}}} \right\rangle$ in the physical basis; $\frac{1}{{\sqrt 2 }}\left\{ {\left| 0 \right\rangle + \left| 1 \right\rangle } \right\} \otimes \left| 0 \right\rangle$, obtained by first rotating the polarization by 45°: $\left| {\rm{V}} \right\rangle$ → $\left| {{{\rm{D}}^{\rm{ + }}}} \right\rangle$ = $\frac{1}{{\sqrt 2 }}\left\{ {\left| {\rm{V}} \right\rangle + \left| {\rm{H}} \right\rangle } \right\}$ (the Hadamard gate *H* in Fig. 5); and $\left| {10} \right\rangle$ corresponding to the state $\left| {{\rm{He}}} \right\rangle$, which is prepared by rotating the polarization $\left| {\rm{V}} \right\rangle$ → $\left| {\rm{H}} \right\rangle$ (the Pauli-*X* operator in Fig. 5).

The measurement results are presented in Fig. 5 for the nine different combinations of selected gate and input state. In each setting, the predictions are borne out by reconstructing the one-photon two-qubit density operator from quantum-state-tomography measurements^50, 51^ in polarization and parity^42, 52^ (via SLM_2_ in Fig. 4; Supplementary Note 3). In the case of the identity gate (*θ* = 0), the input states emerge with no change. The measured density matrices therefore correspond to $\left| {{\psi _1}} \right\rangle = \left| {{\rm{Ve}}} \right\rangle \left\langle {{\rm{Ve}}} \right|$, $\left| {{\psi _2}} \right\rangle = \left| {{{\rm{D}}^ + }{\rm{e}}} \right\rangle \left\langle {{{\rm{D}}^ + }{\rm{e}}} \right|$, and $\left| {{\psi _3}} \right\rangle = \left| {{\rm{He}}} \right\rangle \left\langle {{\rm{He}}} \right|$. When we set *θ* = *π*, we obtain a CNOT gate—a controlled Pauli *X*-operator on the *x*-parity qubit. Therefore, the input state $\left| {00} \right\rangle$ emerges unaffected $\left| {{\psi _1}} \right\rangle$ = $\left| {00} \right\rangle$, $\left| {10} \right\rangle$ is changed to $\left| {{\psi _3}} \right\rangle$ = $\left| {11} \right\rangle$, while the input state $\frac{1}{{\sqrt 2 }}\left\{ {\left| 0 \right\rangle + \left| 1 \right\rangle } \right\} \otimes \left| 0 \right\rangle$ (a superposition of the previous two states $\left| {00} \right\rangle$ and $\left| {10} \right\rangle$) entangles polarization with *x*-parity, producing the maximally entangled Bell state $\left| {{\psi _2}} \right\rangle$ = $\frac{1}{{\sqrt 2 }}\left\{ {\left| {00} \right\rangle + \left| {11} \right\rangle } \right\}$. Finally, setting *θ* = $\frac{\pi }{2}$, we obtain a $\sqrt {{\rm{CNOT}}}$ gate. The performance of these gates is quantified via their fidelity^53^ defined as $F = {\left( {{\rm{Tr}}\left[ {\sqrt {\sqrt \rho \sigma \sqrt \rho } } \right]} \right)^2}$, where “Tr” refers to the trace of an operator, and *ρ* and *σ* are the measured and expected density matrices, respectively, for the states produced in the different configurations. For *θ* = 0, we obtain *F* = 0.9565 ± 0.0010, 0.8984 ± 0.0015, 0.9682 ± 0.0003 for the three input states tested; for *θ* = $\frac{\pi }{2}$, we obtain *F* = 0.9581 ± 0.0008, 0.8851 ± 0.0018, 0.9274 ± 0.0009; and for *θ* = *π*, we obtain *F* = 0.9644 ± 0.0008, 0.8812 ± 0.0019, 0.8981 ± 0.0021.

### Experimental demonstration of three-qubit SPQL

We proceed to describe our results on constructing three-qubit SPQL, with polarization as the control qubit and *x*- and *y*-parity as the target qubits. Crucially, because operations on *x*- and *y*-parity commute, they may be implemented simultaneously on the same SLM by adding the corresponding phases. If a phase factor ${e^{i{\varphi _1}(x)}}$ is required to implement the one-qubit *x*-parity gate $U_1^{(x)}$ and ${e^{i{\varphi _2}(y)}}$ for the *y*-parity gate $U_1^{(y)}$, then the phase factor ${e^{i\left\{ {{\varphi _1}\left( x \right) + {\varphi _2}(y)} \right\}}}$ corresponds to the two-qubit transformation $U_1^{(x)} \otimes U_1^{(y)}$. Furthermore, non-separable phase distributions, *φ*(*x*, *y*) ≠ *φ*_1_(*x*) + *φ*_2_(*y*) such that ${e^{i\varphi \left( {x,y} \right)}} \ne \, {e^{i{\varphi _1}\left( x \right)}}{e^{i{\varphi _2}(y)}}$, can entangle the qubits associated with *x*- and *y*-parity^40^. Care must be exercised in selecting *φ*(*x*, *y*) to guarantee that the parity state-space remains closed under all such transformations. We have shown theoretically that 2D phase distributions that are piecewise constant in the four quadrants satisfy this requirement^34^. Therefore, a polarization-selective SLM can implement a broad range of three-qubit quantum gates with the appropriate selection of the phases in its four quadrants.

Three-qubit states in the Hilbert space of polarization and *xy*-parity are spanned by the basis $\left\{ {\left| {\rm{V}} \right\rangle ,\left| {\rm{H}} \right\rangle } \right\} \otimes {\left\{ {\left| {\rm{e}} \right\rangle ,\left| {\rm{o}} \right\rangle } \right\}_x} \otimes {\left\{ {\left| {\rm{e}} \right\rangle ,\left| {\rm{o}} \right\rangle } \right\}_y}$, and we use a contracted notation: for example $\left| {\rm{V}} \right\rangle \otimes {\left| {\rm{e}} \right\rangle _x}{\left| {\rm{o}} \right\rangle _y} = \left| {{\rm{Veo}}} \right\rangle$—corresponding to $\left| {001} \right\rangle$ in the logical basis. The phase distribution imparted to the photon by the polarization-sensitive SLM (PS-SLM in Fig. 4) rotates the two parity qubits when the control qubit is $\left| {\rm{H}} \right\rangle$. This operator is represented by the matrix

2 $${U_3} = \left( {\begin{array}{*{20}{c}}{{{\Bbb I}_4}} & {{{\bf{0}}_4}} \\ {{{\bf{0}}_4}} & {{R_{xy}}} \end{array}} \right)$$

where ${{\Bbb I}_4}$ and **0**_4_ are the 4D identity and zero operators, respectively, and *R*_*xy*_ is a unitary operator on the 4D space of *xy*-parity^40^.

The three-qubit states utilized in testing such gates are prepared by the heralded single-photon source shown in Fig. 4. When the nonlinear crystal is illuminated by a $\left| {\rm{H}} \right\rangle$-polarized laser whose spatial profile is separable in *x* and *y* and has even-parity along both, then it can be shown that the two-photon state produced is $\left| {\Psi} \right\rangle \propto \left| {{{\rm{V}}_1}{{\rm{V}}_2}} \right\rangle \otimes {\left\{ {\left| {{{\rm{e}}_1}{{\rm{e}}_2}} \right\rangle + \left| {{{\rm{o}}_1}{{\rm{o}}_2}} \right\rangle } \right\}_x} \otimes {\left\{ {\left| {{{\rm{e}}_1}{{\rm{e}}_2}} \right\rangle + \left| {{{\rm{o}}_1}{{\rm{o}}_2}} \right\rangle } \right\}_y}$, where the subscripts 1 and 2 refer to the signal and idler photons, respectively, and the kets are associated with the polarization, *x*-parity, and *y*-parity subspaces^34^. By projecting the idler photon onto a single (even) mode, the heralded photon has the reduced one-photon state $\left| {{\Psi _{\rm{i}}}} \right\rangle$ = $\left| {{\rm{Vee}}} \right\rangle$ in the contracted notation, corresponding to logical $\left| {000} \right\rangle$.

**Fig. 6:**
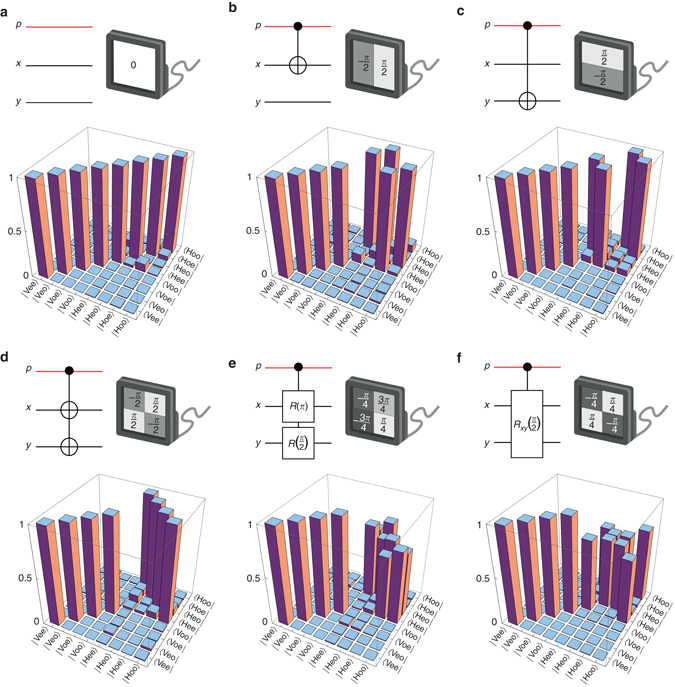
Quantum-circuit representation and the measurement of operators for three-qubit gates. For the quantum gate in each panel, we present the quantum circuit, the 2D SLM phase required for implementing the gate, and the reconstructed transformation operator in the polarization-parity Hilbert space. **a** The identity gate ${{\Bbb I}_x} \otimes {{\Bbb I}_y}$; **b** ${\rm{CNO}}{{\rm{T}}_x} \otimes {{\Bbb I}_y}$; **c** ${{\Bbb I}_x} \otimes {\rm{CNO}}{{\rm{T}}_y}$; **d** ${\rm{CNO}}{{\rm{T}}_x} \otimes {\rm{CNO}}{{\rm{T}}_y}$; **e** a rotation *R*_*x*_(*π*) on the *x*-parity qubit and a rotation *R*_*y*_ $\left( {\frac{\pi }{2}} \right)$ on *y*-parity qubit, corresponding to the separable quantum gate ${\rm{CNO}}{{\rm{T}}_x} \otimes \sqrt {{\rm{CNO}}{{\rm{T}}_y}}$; and **f** a joint rotation *R*_*xy*_ $\left( {\frac{\pi }{2}} \right)$ in the joint Hilbert space of *x*- and *y*-parity

Six three-qubit quantum logic gates are implemented using the SLM (Fig. 6). The operation of each gate is confirmed by generating all eight three-qubit canonical states $\left| {{\rm{Vee}}} \right\rangle$ $\left( {\left| {000} \right\rangle } \right)$ through $\left| {{\rm{Hoo}}} \right\rangle$ $\left( {\left| {111} \right\rangle } \right)$, and then projecting the output state onto this basis to determine the gate’s truth table. Generating the input states requires switching the basis states of the subspaces of polarization ($\left| {\rm{V}} \right\rangle$ → $\left| {\rm{H}} \right\rangle$ via HWP_1_), *x*-parity (${\left| {\rm{e}} \right\rangle _x}$ → ${\left| {\rm{o}} \right\rangle _x}$) and *y*-parity (${\left| {\rm{e}} \right\rangle _y}$ → ${\left| {\rm{o}} \right\rangle _y}$) independently (via SLM_1_ in Fig. 4). For example, starting from $\left| {{\rm{Vee}}} \right\rangle$ we prepare $\left| {{\rm{Heo}}} \right\rangle$ by placing the HWP and a *π* phase-step along *y*, and so on. The three-qubit projections are carried out using a cascade of a PBS and a parity analyzer with the appropriately configured parity prism placed in one arm of a balanced MZI (Supplementary Note 4).

The results for the three-qubit gates are presented in Fig. 6. The phases for the four quadrants implemented by the SLM start from the top right quadrant and move in the counter-clockwise direction. The gates include: unity gate (Fig. 6a) implemented with zero-phase on the SLM; CNOT_*x*_ gate on the *x*-parity qubit utilizing a *π* phase-step along *x* (phases are $\frac{\pi }{2}$, −$\frac{\pi }{2}$, −$\frac{\pi }{2}$, and $\frac{\pi }{2}$; Fig. 6b); CNOT_*y*_ gate on the *y*-parity qubit utilizing a *π* phase-step along *y* (phases are $\frac{\pi }{2}$, $\frac{\pi }{2}$, −$\frac{\pi }{2}$, and −$\frac{\pi }{2}$; Fig. 6c); cascaded CNOT_*x*_ and CNOT_*y*_ gates, or ${\rm{CNO}}{{\rm{T}}_x} \otimes {\rm{CNO}}{{\rm{T}}_y}$, sharing the same control qubit and utilizing the SLM phases $\frac{\pi }{2}$, −$\frac{\pi }{2}$, $\frac{\pi }{2}$, and −$\frac{\pi }{2}$ resulting from adding the phases for the CNOT_*x*_ and CNOT_*y*_ gates and then subtracting an unimportant global phase $\frac{\pi }{2}$ (Fig. 6d); cascaded gates $U_2^{(x)}(\pi ) \otimes U_2^{(y)}\left( {\frac{\pi }{2}} \right)$, corresponding to ${\rm{CNO}}{{\rm{T}}_x} \otimes \sqrt {{\rm{CNO}}{{\rm{T}}_y}}$, implemented using the phases $\frac{{3\pi }}{4}$, −$\frac{\pi }{4}$, −$\frac{{3\pi }}{4}$, and $\frac{\pi }{4}$ resulting from adding the phases for the gates CNOT_*x*_ and $\sqrt {{\rm{CNO}}{{\rm{T}}_y}}$ (Fig. 6e); and finally, a controlled joint rotation of the *xy*-parity implemented using the non-separable phase distribution $\frac{\pi }{4}$, −$\frac{\pi }{4}$, $\frac{\pi }{4}$, and −$\frac{\pi }{4}$ (Fig. 6f). In the latter case, the entangling two-qubit transformation implemented on the *xy*-parity space (when polarization is $\left| {\rm{H}} \right\rangle$) is

3 $${R_{xy}} = \left( {\begin{array}{*{20}{c}}1 & 0 & 0 & i \\ 0 & 1 & i & 0 \\ 0 & i & 1 & 0 \\ i & 0 & 0 & 1 \end{array}} \right)$$

Projections in polarization space are made using a polarization analyzer, and in spatial-parity space using a modified MZI, which acts as the parity analyzer. We have measured the operators, or truth tables and then benchmark the performance of the gates with their “inquisition”^54^, the overlap between the measured ***σ***_m_ and ideal ***σ***_*i*_ matrices defined by ${{\sigma}} I = {\rm{Tr}}\left( {{{\bf{\sigma }}_{\rm{m}}}{\bf{\sigma }}_i^{\rm{T}}} \right){\rm{/Tr}}\left( {{{\bf{\sigma }}_i}{\bf{\sigma }}_i^{\rm{T}}} \right)$, where “T” indicates the conjugate transpose. We find the inquisition to be 0.9986, 0.9967, 0.9974, 0.9975, 0.9977, and 0.9989 (all with average uncertainty of ±0.0010), for the gates depicted in Fig. 6a through Fig. 6f, respectively.

### Generation of single-photon three-qubit GHZ and W states

Finally, as an application of the three-qubit quantum gates described above, we implement entangling gates that convert a generic separable state $\left| {{\rm{Vee}}} \right\rangle$ (logical $\left| {000} \right\rangle$) into entangled states. It is well-known that there are two classes of entangled three-qubit states that cannot be interconverted into each other through local operations: GHZ states such as $\frac{1}{{\sqrt 2 }}\left\{ {\left| {000} \right\rangle + \left| {111} \right\rangle } \right\}$, and W states such as $\frac{1}{{\sqrt 3 }}\left\{ {\left| {001} \right\rangle + \left| {010} \right\rangle + \left| {100} \right\rangle } \right\}$. In our context of single-photon three-qubit states, these two classes of entanglement cannot be interconverted through operations that affect any of the DoFs separately.

**Fig. 7:**
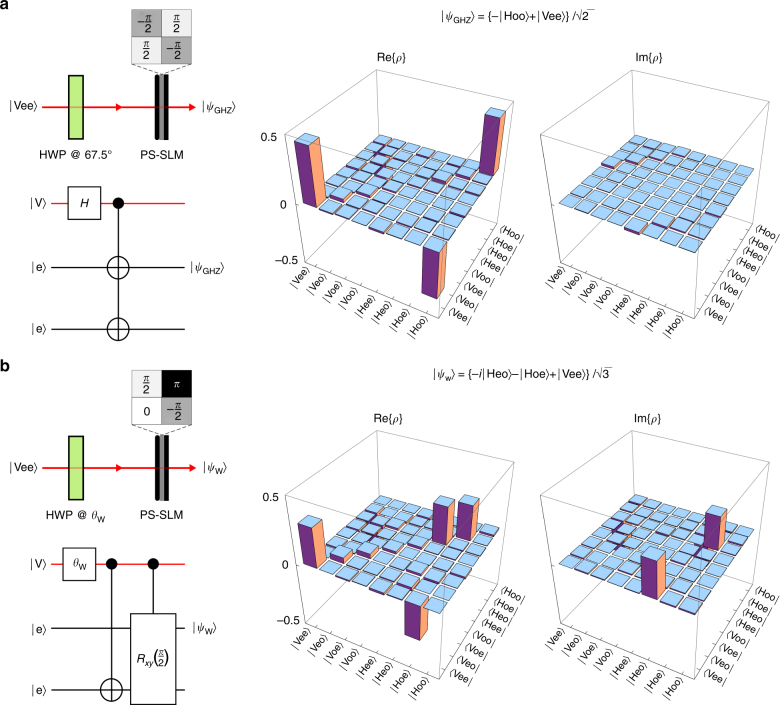
Producing entangled three-qubit states. **a** Implementation and quantum circuit for producing a GHZ state from an initially separable generic state. On the right we plot the real and imaginary parts of the three-qubit density operator *ρ* reconstructed from quantum state tomography measurements. **b** Same as **a** to produce a W state

To prepare a GHZ state starting from the separable state $\left| {{\rm{Vee}}} \right\rangle$, we first rotate the polarization 45°, $\left| {\rm{V}} \right\rangle$ → $\frac{1}{{\sqrt 2 }}\left\{ {\left| {\rm{V}} \right\rangle + \left| {\rm{H}} \right\rangle } \right\}$, and then implement the three-qubit quantum gate shown in Fig. 6d. This gate combines two two-qubit gates: a CNOT gate on *x*-parity and a CNOT gate on *y*-parity—both controlled by the polarization qubit. The SLM imparts alternating phases of $\frac{\pi }{2}$ and −$\frac{\pi }{2}$ in the four quadrants (Fig. 7a). Thus, when the polarization is $\left| {\rm{H}} \right\rangle$, the *x*-parity CNOT gate implements the transformation $\left| {\rm{e}} \right\rangle$ → *i* $\left| {\rm{o}} \right\rangle$ (Eq. (1) with *θ* set to *π*); and similarly for the *y*-parity CNOT gate. Consequently, the resulting three-qubit state evolution is $\left| {{\rm{Vee}}} \right\rangle$ → $\frac{1}{{\sqrt 2 }}\left\{ {\left| {{\rm{Vee}}} \right\rangle + \left| {{\rm{Hee}}} \right\rangle } \right\}$ → $\frac{1}{{\sqrt 2 }}\left\{ {\left| {{\rm{Vee}}} \right\rangle - \left| {{\rm{Hoo}}} \right\rangle } \right\}$, corresponding to a maximally entangled GHZ state. By reconstruction the three-qubit density matrix of the generated state via quantum state tomography, the fidelity is estimated to be 0.8207 ± 0.0027 (Fig. 7a).

To produce a three-qubit W state starting from the same generic separable state $\left| {{\rm{Vee}}} \right\rangle$, we rotate the polarization an angle *φ*_W_, where ${\rm{tan}}\,{\varphi _{\rm{W}}} = \frac{1}{{\sqrt 2 }}$; $\left| {\rm{V}} \right\rangle \to \frac{1}{{\sqrt 3 }}\left\{ {\left| {\rm{V}} \right\rangle + \sqrt 2 \left| {\rm{H}} \right\rangle } \right\}$. The SLM imparts the phases *π*, $\frac{\pi }{2}$, 0, and −$\frac{\pi }{2}$ in the four quadrants. The phase modulation is non-separable along *x* and *y*, and thus imparts an entangling rotation in the space of *x*- and *y*-parity. Specifically, when the polarization is $\left| {\rm{H}} \right\rangle$, the parity is mapped according to $\left| {{\rm{ee}}} \right\rangle$ → $\frac{1}{{\sqrt 2 }}\left\{ {i\left| {{\rm{eo}}} \right\rangle - \left| {{\rm{oe}}} \right\rangle } \right\}$. Consequently, the resulting three-qubit state evolution is $\left| {{\rm{Vee}}} \right\rangle$ → $\frac{1}{{\sqrt 3 }}\left\{ {\left| {{\rm{Vee}}} \right\rangle + \sqrt 2 \left| {{\rm{Hee}}} \right\rangle } \right\}$ → $\frac{1}{{\sqrt 3 }}\left\{ {\left| {{\rm{Vee}}} \right\rangle + i\left| {{\rm{Heo}}} \right\rangle - \left| {{\rm{Hoe}}} \right\rangle } \right\}$, corresponding to a maximally entangled W state. The fidelity of the reconstructed W state estimated through quantum state tomography is 0.8284 ± 0.0026 (Fig. 7b).

## Discussion

In conclusion, we have experimentally demonstrated linear, deterministic, single-photon, two- and three-qubit quantum logic gates using polarization and spatial parity qubits that are implemented by a single optical device, a polarization-selective SLM. The average fidelity for two-qubit SPQL is 93%, whereas that for three-qubit SPQL is 83%. The performance of these gates is limited essentially by two factors: the quantization of the phase implemented on the SLM and its diffraction efficiency, both of which are expected to be reduced with advancements in SLM technology. Another factor that reduces the fidelities of the measured states is the alignment of the interferometers utilized in parity analysis, which provide a baseline visibility of ~94% and thus lead to an underestimation of the fidelities. This imperfection can be obviated by implementing active control in the interferometer.

The advantages of the spatial-parity-encoding scheme we described in the introduction and demonstrated in our experiments have all been confirmed for the case of two qubits encoded in the 2D transverse spatial profile of single-photon states in a Cartesian coordinate system. In alternative encoding techniques, such as those associated with OAM states, a higher-dimensionality Hilbert space is accessible in the azimuthal DOF (in a polar coordinate system), by utilizing high-order modes. A comparable approach can be exploited in our scheme, where the 2D transverse plane is segmented into non-overlapping square areas where the spatial parity qubits are encoded independently. This would allow us to increase the number of qubits per photon with the same SLM-based modulation scheme—at the price, however, of increasing the complexity of the detection system. Increasing the number of qubits per photon can be exploited in few-qubit applications such as quantum communications where the redundancy resulting from embedding a logical state in a larger-dimension Hilbert space can help combat decoherence.

In this work, we have shown how three qubits can be encoded in the polarization and spatial parity DoFs of a single photon. Instead of heralding the arrival of one photon from a pair of entangled photons by detecting the second photon, the two photons in the pair may both be exploited^34^. In this scenario, the two-photon state can be used to encode six qubits, and each set of three qubits (for each photon) is readily manipulated with an SLM. The use of both photons opens up a host of interesting possibilities, such as the creation of six-qubit cluster states, production of exotic hyper-entangled states, and tests of quantum nonlocality^55–58^.

Finally, this approach may also be applied to other DoFs, such as OAM, to realize quantum gates in which the polarization qubit acts as the control and the OAM qubit as the target. In contrast with OAM states, the appealing features of spatial parity include the non-necessity of truncation of Hilbert space via modal filtering of photons using slits or pinholes, and the simplicity of constructing operators in spatial-parity space. Multiple gates may be readily cascaded, thereby paving the way to convenient implementations of few-qubit quantum information processing algorithms.

## Methods

### Experimental setup

We produce photon pairs by type-I collinear SPDC when an $\left| {\rm{H}} \right\rangle$-polarized monochromatic pump laser with an even spatial profile from a diode laser (Coherent CUBE 405-50, 405 nm, 50 mW) impinges on a 1.5-mm-thick β-barium-borate (BBO) crystal propagating at an angle of 28.3° from the crystal axis. The pump is subsequently removed using a Glan-Thompson polarizer and a 10-nm-bandwidth interference filter centered at 810 nm. One of the two $\left| {\rm{V}} \right\rangle$-polarized photons heralds the arrival of the other by coupling through a single-mode fiber (SMF) to a single-photon-sensitive avalanche photodiode, APD (PerkinElmer SPCM-AQR). The heralded photons after the setup are collected through a multimode fiber to another APD.

The setup divides into three stages: state preparation, control, and analysis. Coupling the trigger photon into a SMF projects the heralded photon onto a single, even-parity spatial mode, such that its state may be written as $\left| \Psi \right\rangle$ = $\left| {{\rm{He}}} \right\rangle$. This state may be further modified using a sequence of a SLM (SLM_1_ to prepare the parity state) and a HWP (to rotate the polarization). The state control is implemented using the controlled-unitary quantum gate realized with a polarization sensitive SLM (SLM_2_). In the case of *x*-parity, we use a step phase pattern (*θ*) on SLM_2_ along *x*; similarly for *y*-parity. State analysis cascades a polarization projection (a HWP and a PBS) followed by a parity projection (SLM_3_ and a MZI); see Supplementary Notes 2 and 3 for the configurations used in the two-qubit and three-qubit SPQL experiments, respectively.

See Supplementary Methods for details on data acquisition, SLM calibration, and alignment protocols.

### Three-qubit tomography in an alternate basis

A key step in the quantum state tomography used in the main text is first finding the multi-DoF Stokes parameters^51, 52, 59^, which are then used to estimate the density matrix^42^. The actual settings for the combined polarization and spatial-parity projections are directly related to intermediary parameters (denoted as *T*_*j*_, *j* = 1…64), which are then transformed into the Stokes parameters via:

4 $${\left( {\begin{array}{*{20}{c}} {{T_1}} \\ {{T_2}} \\ {{T_3}} \\ {{T_4}} \\ . \\ . \\ . \\ . \\ {{T_{61}}} \\ {{T_{62}}} \\ {{T_{63}}} \\ {{T_{64}}} \\ \end{array}} \right) = \left( {\begin{array}{*{20}{c}}{{\rm{Tr}}\left\{ {{\tau _1}{\sigma _{000}}} \right\}} & {{\rm{Tr}}\left\{ {{\tau _1}{\sigma _{001}}} \right\}} & {{\rm{Tr}}\left\{ {{\tau _1}{\sigma _{002}}} \right\}} & {{\rm{Tr}}\left\{ {{\tau _1}{\sigma _{003}}} \right\}} & . & . & . & . & {{\rm{Tr}}\left\{ {{\tau _1}{\sigma _{330}}} \right\}} & {{\rm{Tr}}\left\{ {{\tau _1}{\sigma _{331}}} \right\}} & {{\rm{Tr}}\left\{ {{\tau _1}{\sigma _{332}}} \right\}} & {{\rm{Tr}}\left\{ {{\tau _1}{\sigma _{333}}} \right\}} \\ {{\rm{Tr}}\left\{ {{\tau _2}{\sigma _{000}}} \right\}} & {{\rm{Tr}}\left\{ {{\tau _2}{\sigma _{001}}} \right\}} & {{\rm{Tr}}\left\{ {{\tau _2}{\sigma _{002}}} \right\}} & {{\rm{Tr}}\left\{ {{\tau _2}{\sigma _{003}}} \right\}} & . & . & . & . & {{\rm{Tr}}\left\{ {{\tau _2}{\sigma _{330}}} \right\}} & {{\rm{Tr}}\left\{ {{\tau _2}{\sigma _{331}}} \right\}} & {{\rm{Tr}}\left\{ {{\tau _2}{\sigma _{332}}} \right\}} & {{\rm{Tr}}\left\{ {{\tau _2}{\sigma _{333}}} \right\}} \\ {{\rm{Tr}}\left\{ {{\tau _3}{\sigma _{000}}} \right\}} & {{\rm{Tr}}\left\{ {{\tau _3}{\sigma _{001}}} \right\}} & {{\rm{Tr}}\left\{ {{\tau _3}{\sigma _{002}}} \right\}} & {{\rm{Tr}}\left\{ {{\tau _3}{\sigma _{003}}} \right\}} & . & . & . & . & {{\rm{Tr}}\left\{ {{\tau _3}{\sigma _{330}}} \right\}} & {{\rm{Tr}}\left\{ {{\tau _3}{\sigma _{331}}} \right\}} & {{\rm{Tr}}\left\{ {{\tau _3}{\sigma _{332}}} \right\}} & {{\rm{Tr}}\left\{ {{\tau _3}{\sigma _{333}}} \right\}} \\ {{\rm{Tr}}\left\{ {{\tau _4}{\sigma _{000}}} \right\}} & {{\rm{Tr}}\left\{ {{\tau _4}{\sigma _{001}}} \right\}} & {{\rm{Tr}}\left\{ {{\tau _4}{\sigma _{002}}} \right\}} & {{\rm{Tr}}\left\{ {{\tau _4}{\sigma _{003}}} \right\}} & . & . & . & . & {{\rm{Tr}}\left\{ {{\tau _4}{\sigma _{330}}} \right\}} & {{\rm{Tr}}\left\{ {{\tau _4}{\sigma _{331}}} \right\}} & {{\rm{Tr}}\left\{ {{\tau _4}{\sigma _{332}}} \right\}} & {{\rm{Tr}}\left\{ {{\tau _4}{\sigma _{333}}} \right\}} \\ . & . & . & . & . & . & . & . & . & . & . & . \\ . & . & . & . & . & . & . & . & . & . & . & . \\ . & . & . & . & . & . & . & . & . & . & . & . \\ . & . & . & . & . & . & . & . & . & . & . & . \\ {{\rm{Tr}}\left\{ {{\tau _{61}}{\sigma _{000}}} \right\}} & {{\rm{Tr}}\left\{ {{\tau _{61}}{\sigma _{001}}} \right\}} & {{\rm{Tr}}\left\{ {{\tau _{61}}{\sigma _{002}}} \right\}} & {{\rm{Tr}}\left\{ {{\tau _{61}}{\sigma _{003}}} \right\}} & . & . & . & . & {{\rm{Tr}}\left\{ {{\tau _{61}}{\sigma _{330}}} \right\}} & {{\rm{Tr}}\left\{ {{\tau _{61}}{\sigma _{331}}} \right\}} & {{\rm{Tr}}\left\{ {{\tau _{61}}{\sigma _{332}}} \right\}} & {{\rm{Tr}}\left\{ {{\tau _{61}}{\sigma _{333}}} \right\}} \\ {{\rm{Tr}}\left\{ {{\tau _{62}}{\sigma _{000}}} \right\}} & {{\rm{Tr}}\left\{ {{\tau _{62}}{\sigma _{001}}} \right\}} & {{\rm{Tr}}\left\{ {{\tau _{62}}{\sigma _{002}}} \right\}} & {{\rm{Tr}}\left\{ {{\tau _{62}}{\sigma _{003}}} \right\}} & . & . & . & . & {{\rm{Tr}}\left\{ {{\tau _{62}}{\sigma _{330}}} \right\}} & {{\rm{Tr}}\left\{ {{\tau _{62}}{\sigma _{331}}} \right\}} & {{\rm{Tr}}\left\{ {{\tau _{62}}{\sigma _{332}}} \right\}} & {{\rm{Tr}}\left\{ {{\tau _{62}}{\sigma _{333}}} \right\}} \\ {{\rm{Tr}}\left\{ {{\tau _{63}}{\sigma _{000}}} \right\}} & {{\rm{Tr}}\left\{ {{\tau _{63}}{\sigma _{001}}} \right\}} & {{\rm{Tr}}\left\{ {{\tau _{63}}{\sigma _{002}}} \right\}} & {{\rm{Tr}}\left\{ {{\tau _{63}}{\sigma _{003}}} \right\}} & . & . & . & . & {{\rm{Tr}}\left\{ {{\tau _{63}}{\sigma _{330}}} \right\}} & {{\rm{Tr}}\left\{ {{\tau _{63}}{\sigma _{331}}} \right\}} & {{\rm{Tr}}\left\{ {{\tau _{63}}{\sigma _{332}}} \right\}} & {{\rm{Tr}}\left\{ {{\tau _{63}}{\sigma _{333}}} \right\}} \\ {{\rm{Tr}}\left\{ {{\tau _{64}}{\sigma _{000}}} \right\}} & {{\rm{Tr}}\left\{ {{\tau _{64}}{\sigma _{001}}} \right\}} & {{\rm{Tr}}\left\{ {{\tau _{64}}{\sigma _{002}}} \right\}} & {{\rm{Tr}}\left\{ {{\tau _{64}}{\sigma _{003}}} \right\}} & . & . & . & . & {{\rm{Tr}}\left\{ {{\tau _{64}}{\sigma _{330}}} \right\}} & {{\rm{Tr}}\left\{ {{\tau _{64}}{\sigma _{331}}} \right\}} & {{\rm{Tr}}\left\{ {{\tau _{64}}{\sigma _{332}}} \right\}} & {{\rm{Tr}}\left\{ {{\tau _{64}}{\sigma _{333}}} \right\}} \\ \end{array}} \right)\left( {\begin{array}{*{20}{c}}{{S_{000}}} \\ {{S_{001}}} \\ {{S_{002}}} \\ {{S_{003}}} \\ . \\ . \\ . \\ . \\ {{S_{330}}} \\ {{S_{331}}} \\ {{S_{332}}} \\ {{S_{333}}} \end{array}} \right)}$$

The operators *τ*_*j*_, *j* = 1…64, are each separable in the subspaces of polarization, *x*-parity, and *y*-parity, *τ*_*j*_ = *A*_P_ ⊗ *B*_*x*_ ⊗ *C*_*y*_. The operators *τ*_01_ through *τ*_16_ have the explicit form (each having the same operator on the polarization subspace):

$${\begin{array}{ccccc}\\ &{\tau _{01}} =  \frac{1}{2}\left\{ {{\Bbb I} + Z} \right\} \otimes {\Bbb I} \otimes {\Bbb I}{\rm{;}} \,\, {\tau _{02}} = \frac{1}{2}\left\{ {{\Bbb I} + Z} \right\} \otimes Z \otimes {\Bbb I}; \,\, {\tau _{03}} = \frac{1}{2}\left\{ {{\Bbb I} + Z} \right\} \otimes Y \otimes {\Bbb I};\\ & {\tau _{04}} = \frac{1}{2}\left\{ {{\Bbb I} + Z} \right\} \otimes Y \otimes X  \hskip17.4pc \\ &{\tau _{05}} =  \frac{1}{2}\left\{ {{\Bbb I} + Z} \right\} \otimes {\Bbb I} \otimes Z; \,\, {\tau _{06}} = \frac{1}{2}\left\{ {{\Bbb I} + Z} \right\} \otimes {\Bbb I} \otimes Y; \,\, {\tau _{07}} = \frac{1}{2}\left\{ {{\Bbb I} + Z} \right\} \otimes X \otimes Y;\\  & {\tau _{08}} = \frac{1}{2}\left\{ {{\Bbb I} + Z} \right\} \otimes Z \otimes Z  \hskip17.8pc \\ &{\tau _{09}} = \frac{1}{2}\left\{ {{\Bbb I} + Z} \right\} \otimes Z \otimes Y; \,\, {\tau _{10}} = \frac{1}{2}\left\{ {{\Bbb I} + Z} \right\} \otimes Y \otimes Z; \,\, {\tau _{11}} = \frac{1}{2}\left\{ {{\Bbb I} + Z} \right\} \otimes Y \otimes Y; \\ & {\tau _{12}} = \frac{1}{2}\left\{ {{\Bbb I} + Z} \right\} \otimes X \otimes {\Bbb I}  \hskip17.8pc \\ & {\tau _{13}} = \frac{1}{2}\left\{ {{\Bbb I} + Z} \right\} \otimes {\Bbb I} \otimes X; \,\, {\tau _{14}} = \frac{1}{2}\left\{ {{\Bbb I} + Z} \right\} \otimes X \otimes X; \,\, {\tau _{15}} = \frac{1}{2}\left\{ {{\Bbb I} + Z} \right\} \otimes Z \otimes X; \\ & {\tau _{16}} = \frac{1}{2}\left\{ {{\Bbb I} + Z} \right\} \otimes X \otimes Z \hskip17.4pc \end{array}}$$

The operators *τ*_17_ through *τ*_32_ have the same form except that the polarization operator $\frac{1}{2}\left\{ {{\Bbb I} + Z} \right\}$ is replaced by $\frac{1}{2}\left\{ {{\Bbb I} - Z} \right\}$; for operators *τ*_33_ through *τ*_48_ it is replaced by ${\Bbb I} + X$; and for operators *τ*_33_ through *τ*_48_ it is replaced by ${\Bbb I} + Y$.

### Data availability

The data that support the findings of this study are available from the corresponding author on reasonable request.

## Electronic supplementary material

Supplementary Information
