# Supplementary material for: Single-photon three-qubit quantum logic using spatial light modulators
Source: Nat Commun. 2017 Sep 29;8:739. doi: 10.1038/s41467-017-00580-x (PMC5622142; doi:10.1038/s41467-017-00580-x)
Supplement: Supplementary file 1 — Supplementary Information [file 41467_2017_580_MOESM1_ESM.docx]

**Supplementary Note 1 | Parity prism**


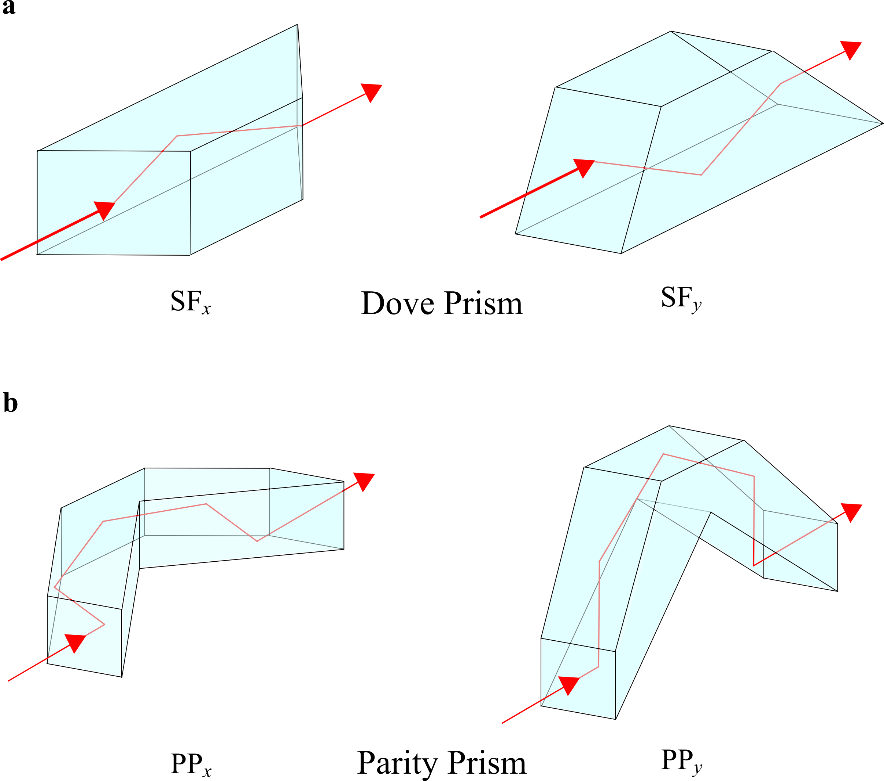


**Supplementary Figure 1 | Comparison of a Dove prism and a parity prism.** **a**, Beam path in a Dove prism oriented to flip a beam along $x$ and $y$: SF*_x_* (left) and SF*_y_* (right), respectively. **b**, Beam path in a parity prism oriented to flip a beam along $x$ and $y$: PP*_x_* (left) and PP*_y_* (right), respectively. SF: spatial flipper; PP: parity prism.

As described in the main text, implementation of a ‘parity analyzer’ – a device that separates the even- and odd-modal content of an optical beam into two paths – requires modifying a balanced Mach-Zehnder interferometer (MZI). This modification consists of placing a ‘spatial flipper’ in one of the MZI arms. The purpose of the spatial flipper is to invert (or flip) the transverse spatial profile of an optical beam along $x$ or $y$. It is critical for the successful operation of parity analysis that the spatial flipper be transparent with respect to polarization, such that the parity analyzer operates solely on the parity Hilbert space and is independent of the state of polarization.

The task of spatial flipping has been traditionally performed with a Dove prism, which is a single-refraction prism capable of producing flips along $x$ or $y$ when oriented appropriately. However, the entrance and exit facets of a Dove prism are inclined with respect to the propagation axis, therefore restricting its utility to only collimated beams. Crucially, transmission through a Dove prism introduces polarization-dependent losses at the entrance and exit facets and at the refraction interface if not provided with an anti-reflection coating. Furthermore, it is well-known that a Dove prism also introduces a change in the state of polarization of the transmitted beam [1, 2], which has an undesirable effect on the assessment of the operation of the quantum logic gates by reducing the estimated state fidelity.

To overcome these drawbacks, we have designed a special prism, which we refer to as a ‘parity prism’ (Supplementary Figs. 1 and 2). The parity prism was custom-manufactured by Optimax Systems, Inc. The image rotation in a parity prism is based on five total-internal-reflections. The entrance and exit facets of a parity prism are normal to the beam, therefore making it less prone to polarization-dependent losses. The parity prism can be used with a converging or diverging beam, as is the case for spontaneous parametric down-conversion produced in a divergent cone in our experiment. Moreover, only the entrance and exit facets are anti-reflection-coated for optimal performance, and the five refracting surfaces can be left uncoated. Another feature of the parity prism is its high beam-pointing stability under rotation, which is particularly useful since the prism is used in different orientations for introducing *x* and *y* flips without comprising the alignment. Finally, to compensate for the path length and loss introduced by the parity prism in one arm of the parity analyzer, we place an antireflection-coated parallelepiped of a similar length and cross-section in the other arm.


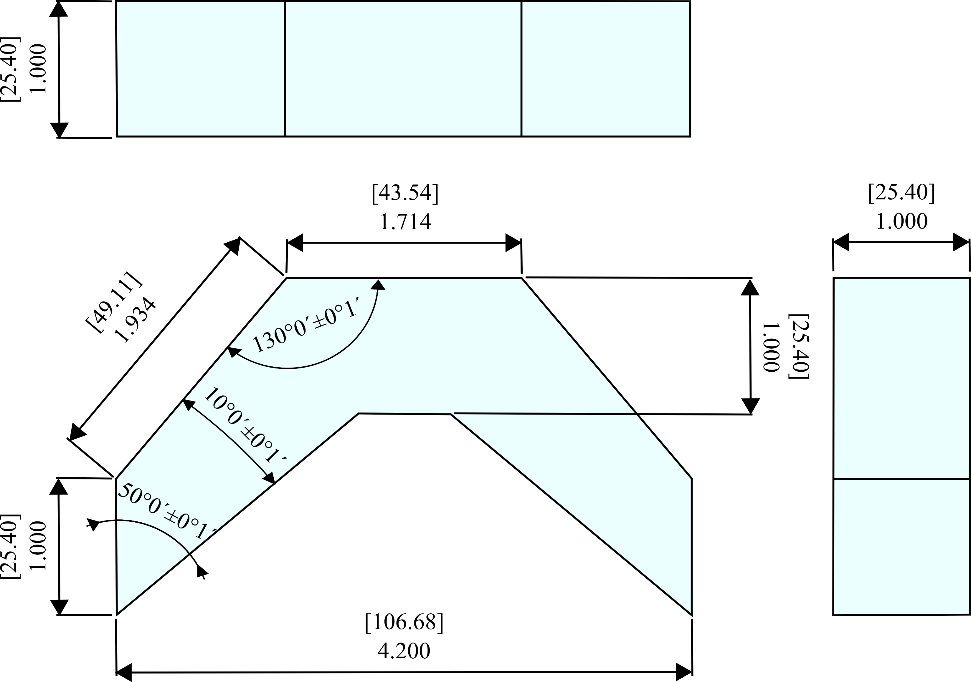


**Supplementary Figure 2 |** **Parity prism dimensions.** Top, front, and side views of the parity prism. Linear dimensions are in inches [millimeters], and angular dimensions are in degrees and arc-minutes.

**Supplementary Note 2 | Setup**

We present here a more detailed description of the optical setup used in our experiments to augment the schematic outline provided in Fig. 4 of the main text. Supplementary Fig. 3 depicts a three-dimensional rendering of the optical arrangement that corresponds to the actual setup we constructed and used in data collection.


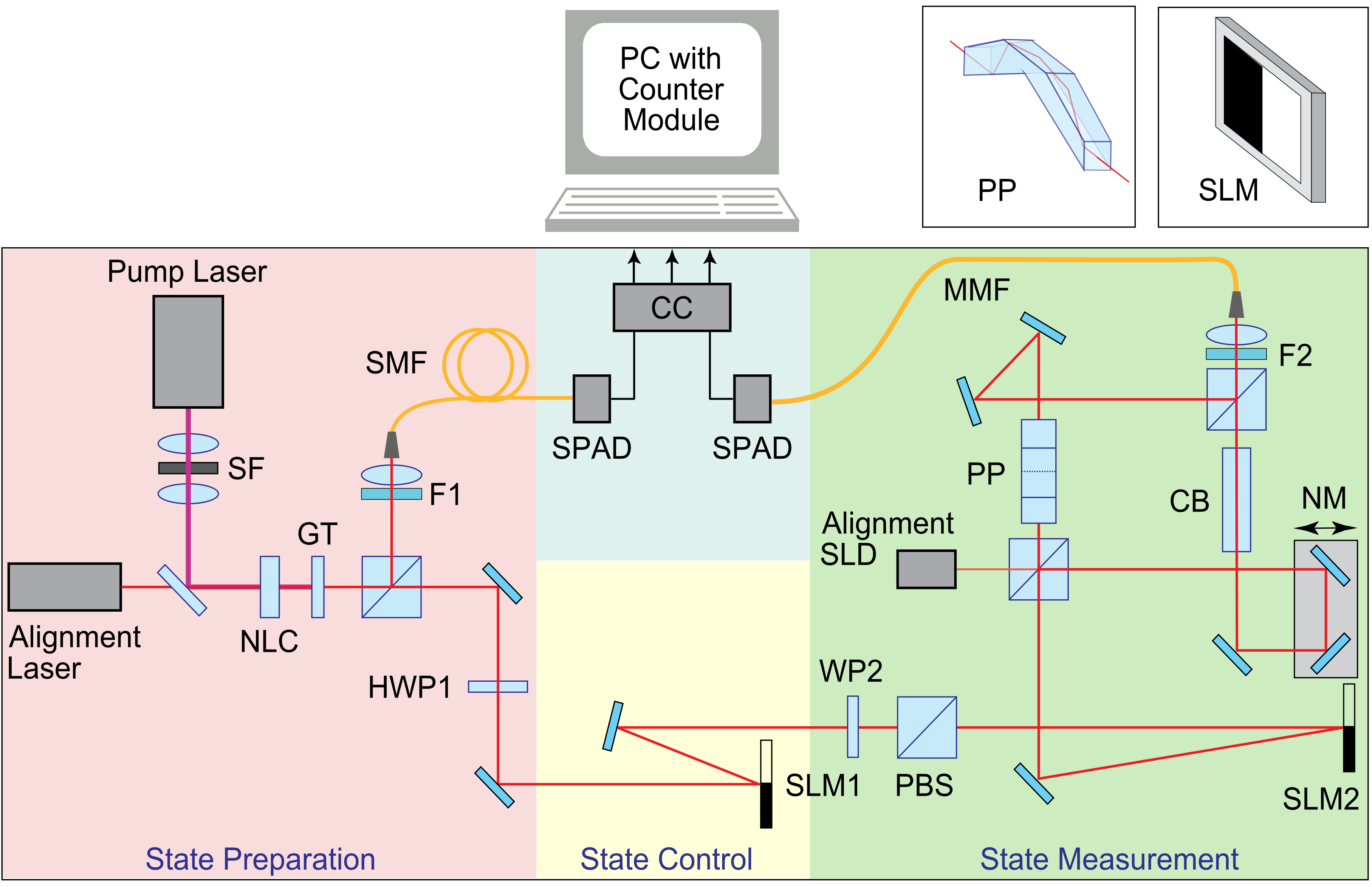


**Supplementary Figure 3 | Detailed experimental setup.** The color of the table-top sections help identify the different stages of the experiment: state preparation (pink); state control via the quantum gate (yellow); state measurement (green); and photon singles and coincidence counting (blue). SF: spatial filter; NLC: nonlinear crystal; GT: Glan-Thomson polarizer; F: interference filter (F1 and F2); SMF: single-mode fiber; HWP: half-wave plate; SLM: spatial light modulator (SLM1 and SLM2); WP: wave plate (either a half-wave plate or a quarter-wave plate depending on the configuration); PBS: polarizing beam splitter; SLD: superluminescent laser diode; PP: parity prism; NM: nano-mover system; CB: compensating block; MMF: multimode fiber; SPAD: single-photon avalanche diode; CC: Coincidence circuitry. Insets show a SLM and the beam path through the parity prism oriented to create a $y$-flip.

**Supplementary Note 3 | Two-qubit projections on joint polarization and *x*-parity space for quantum state tomography**

We express a two-qubit density matrix $\hat{\rho}$ as follows:

$\hat{\rho}=\frac{1}{4}\sum_{j,k=0}^{3} S_{jk}\hat{\sigma}_{j}\bigotimes\hat{\sigma}_{k}$, (1)

where $\left\{ \hat{\sigma}_{j} \right\}$ are the usual Pauli matrices on the subspaces for each of the two qubits,

$\hat{\sigma}_{0}=\left( \begin{matrix} 1 & 0 \\ 0 & 1 \end{matrix} \right)$, $\hat{\sigma}_{1}=\left( \begin{matrix} 0 & 1 \\ 1 & 0 \end{matrix} \right)$, $\hat{\sigma}_{2}=\left( \begin{matrix} 0 & i \\ -i & 0 \end{matrix} \right)$, $\hat{\sigma}_{3}=\left( \begin{matrix} 1 & 0 \\ 0 & -1 \end{matrix} \right)$, (2)

and $\left\{ S_{jk} \right\}$ are two-qubit ‘Stokes parameters’ previously studied in Supplementary Refs. [3-5]. The values for the two-qubit Stokes parameters $S_{jk}$ are determined through

$S_{jk}=\mathrm{Tr}\left\{ \left( \hat{\sigma}_{j}\bigotimes\hat{\sigma}_{k} \right)\hat{\rho} \right\}$, (3)

which can be obtained experimentally via projective measurements carried out in cascade over the sub-spaces associated with each qubit. In our case of a two-qubit state comprising polarization and *x*-parity qubits, such a scheme would take the general form shown in Supplementary Fig. 4. Obtaining $\left\{ S_{jk} \right\}$ then allows us to reconstruct $\hat{\rho}$.


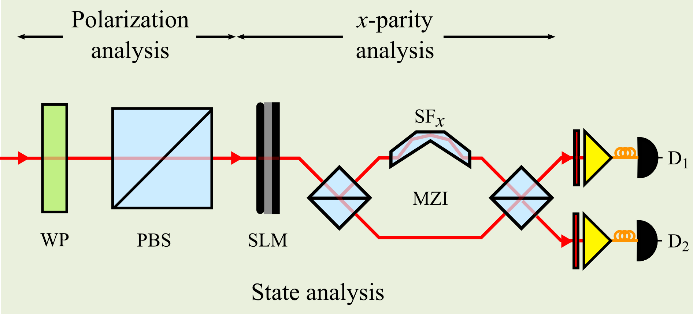


**Supplementary Figure 4 | Two-qubit projective measurements via a cascade of one-qubit projections.** From left to right, projections are carried out on the polarization and $x$-parity spaces. WP: wave-plate; PBS: polarizing beam splitter; SLM: spatial light modulator; SF: spatial flipper implemented by a parity prism operating on the $x$-parity (SF_x_); MZI: Mach-Zehnder interferometer; D_1_ and D_2_: single-photon-sensitive detectors. The SLM here is not polarization sensitive. At the output ports, light is passed through a spectral filter, and then coupled into fibers that deliver the photons to the detectors.

Here, we outline our approach for measuring the 16 two-qubit Stokes parameters $\left\{ S_{jk} \right\}$ required for the analysis of polarization and *x*-parity qubits.

1. $S_{00}$:

Measurement of $S_{00}$ is carried out with the setups shown in Supplementary Fig. 5. The polarization projection consists of a half-wave plate (HWP) with the fast axis oriented at 0° and 45°, followed by a polarizing beamsplitter (PBS), which enables projecting the single-photon state along $|\left. H \right\rangle$ and $|\left. V \right\rangle$, respectively. The parity projection consists of an SLM imparting no phase, followed by a balanced Mach-Zehnder interferometer (MZI) containing in one arm a spatial flipper along $x$ (SF*_x_*). At the output ports we obtain $x$-parity projections along $|\left. e \right\rangle$ and $|\left. o \right\rangle$. By combining the polarization and $x$-parity projections, we implement the following projection operators:

$\hat{\Pi}_{\mathrm{He}}=\left( \begin{matrix} 1 & 0 & 0 & 0 \\ 0 & 0 & 0 & 0 \\ 0 & 0 & 0 & 0 \\ 0 & 0 & 0 & 0 \end{matrix} \right)$, $\hat{\Pi}_{\mathrm{Ho}}=\left( \begin{matrix} 0 & 0 & 0 & 0 \\ 0 & 1 & 0 & 0 \\ 0 & 0 & 0 & 0 \\ 0 & 0 & 0 & 0 \end{matrix} \right)$,

$\hat{\Pi}_{\mathrm{Ve}}=\left( \begin{matrix} 0 & 0 & 0 & 0 \\ 0 & 0 & 0 & 0 \\ 0 & 0 & 1 & 0 \\ 0 & 0 & 0 & 0 \end{matrix} \right)$, $\hat{\Pi}_{\mathrm{Vo}}=\left( \begin{matrix} 0 & 0 & 0 & 0 \\ 0 & 0 & 0 & 0 \\ 0 & 0 & 0 & 0 \\ 0 & 0 & 0 & 1 \end{matrix} \right)$. (4)

Measurements of the probabilities of the four mutually exclusive and exhaustive events $P_{\mathrm{He}}$, $P_{\mathrm{Ho}}$, $P_{\mathrm{Ve}}$, and $P_{\mathrm{Vo}}$ at the outputs yield $S_{00}$: $S_{00}=P_{\mathrm{He}}+P_{\mathrm{Ho}}+P_{\mathrm{Ve}}{+P}_{\mathrm{Vo}}$.


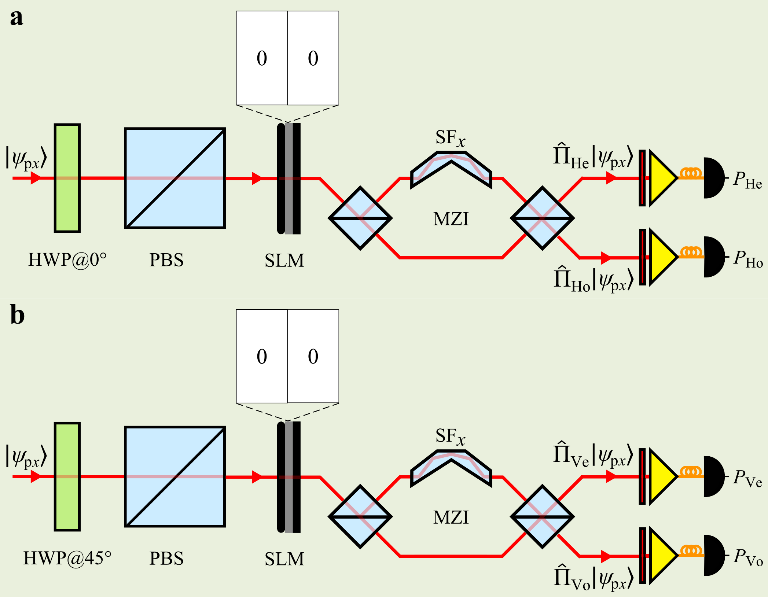


**Supplementary Figure 5 |** **Measurements required to obtain** $\boldsymbol{S}_{\boldsymbol{00}}$**.** **a**, Setup to measure the probabilities $P_{He}$ and $P_{Ho}$ by projecting the polarization along $|\left. H \right\rangle$ and then projecting $|\left. e \right\rangle$ and $|\left. o \right\rangle$. **b**, Same as (**a**) to measure $P_{Ve}$ and $P_{Vo}$.

1. $S_{01}$:

Measurement of $S_{01}$ requires the projections $\hat{\Pi}_{\mathrm{He}}$ and $\hat{\Pi}_{\mathrm{Ve}}$ shown in Supplementary Fig. 5. From these measurements we obtain $S_{01}=2P_{\mathrm{He}}-{2P}_{\mathrm{Ve}}-1$.

1. $S_{02}$:

Measurement of $S_{02}$ is carried out with the setups shown in Supplementary Fig. 6. The polarization projection consists of a HWP with the fast axis oriented at 0° and 45°, followed by a PBS), which enables projecting the single-photon state along $|\left. H \right\rangle$ and $|\left. V \right\rangle$, respectively. The parity projection consists of an SLM that imparts no phase, followed by a ‘beam clipper’ blocking the $-x$ plane half (BC_-_*_x_*). This combination of and SLM and BC projects the $x$-parity onto the parity basis vector $|\left. e+o \right\rangle$. The following projection operators are thus implemented:

$\hat{\Pi}_{H,e+o}=\frac{1}{2}\left( \begin{matrix} 1 & 1 & 0 & 0 \\ 1 & 1 & 0 & 0 \\ 0 & 0 & 0 & 0 \\ 0 & 0 & 0 & 0 \end{matrix} \right)$, $\hat{\Pi}_{V,e+o}=\frac{1}{2}\left( \begin{matrix} 0 & 0 & 0 & 0 \\ 0 & 0 & 0 & 0 \\ 0 & 0 & 1 & 1 \\ 0 & 0 & 1 & 1 \end{matrix} \right)$. (5)

Measurements of $P_{H,e+o}$and $P_{V,e+o}$ yield $S_{02}$: $S_{02}={2P}_{H,e+o}+2P_{V,e+o}-1$.


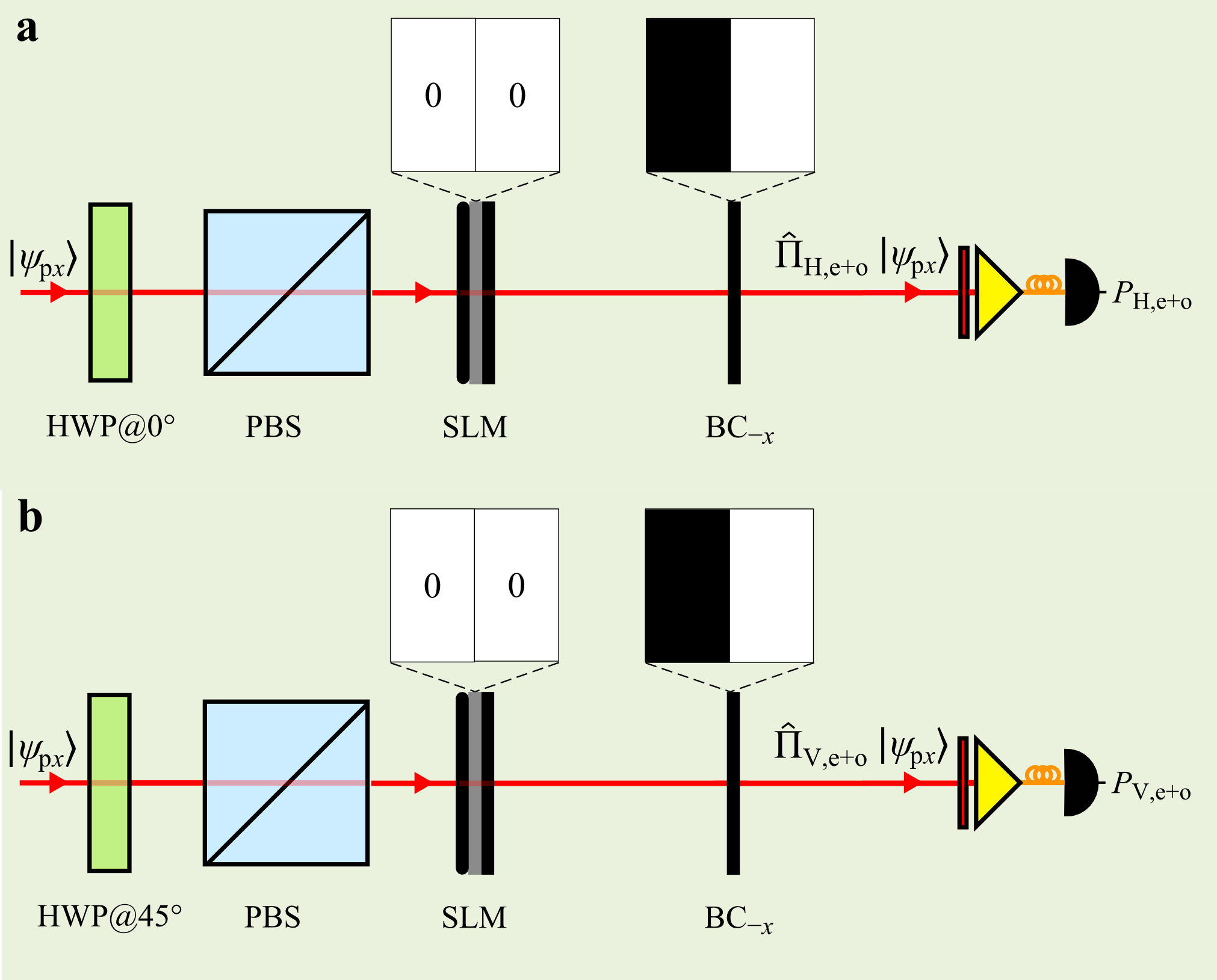


**Supplementary Figure 6 |** **Measurements required to obtain** $\boldsymbol{S}_{\boldsymbol{02}}$**.** **a**, Setup to project the single-photon-state onto the $|\left. H,e+o \right\rangle$ state. **b**, Same as (**a**) to project onto the $|\left. V,e+o \right\rangle$ state. BC_-x_: a ‘beam clipper’ that blocks the $-x$ half plane.

1. $S_{03}$:

Measurement of $S_{03}$ is carried out with the setups shown in Supplementary Fig. 7. The polarization projection consists of a HWP with the fast axis oriented at 0° and 45°, followed by a PBS), which enables projecting the single-photon state along $|\left. H \right\rangle$ and $|\left. V \right\rangle$, respectively. The parity projection along the $|\left. e+io \right\rangle$ state consists of an SLM that imparts a $\frac{\pi}{2}$ phase step along $x$ (hence implementing a $\frac{\pi}{2}$ rotation for the $x$-parity qubit) followed by a MZI containing a spatial flipper along $x$ in one arm. The operators implemented are:

$\hat{\Pi}_{H,e+io}=\frac{1}{2}\left( \begin{matrix} 1 & -i & 0 & 0 \\ i & 1 & 0 & 0 \\ 0 & 0 & 0 & 0 \\ 0 & 0 & 0 & 0 \end{matrix} \right)$, $\hat{\Pi}_{Ve+io}=\frac{1}{2}\left( \begin{matrix} 0 & 0 & 0 & 0 \\ 0 & 0 & 0 & 0 \\ 0 & 0 & 1 & -i \\ 0 & 0 & i & 1 \end{matrix} \right)$. (6)

Measurements $P_{H,e+io}$and $P_{V,e+io}$ yield $S_{03}$: $S_{03}={2P}_{H,e+io}+2P_{V,e+io}-1$.


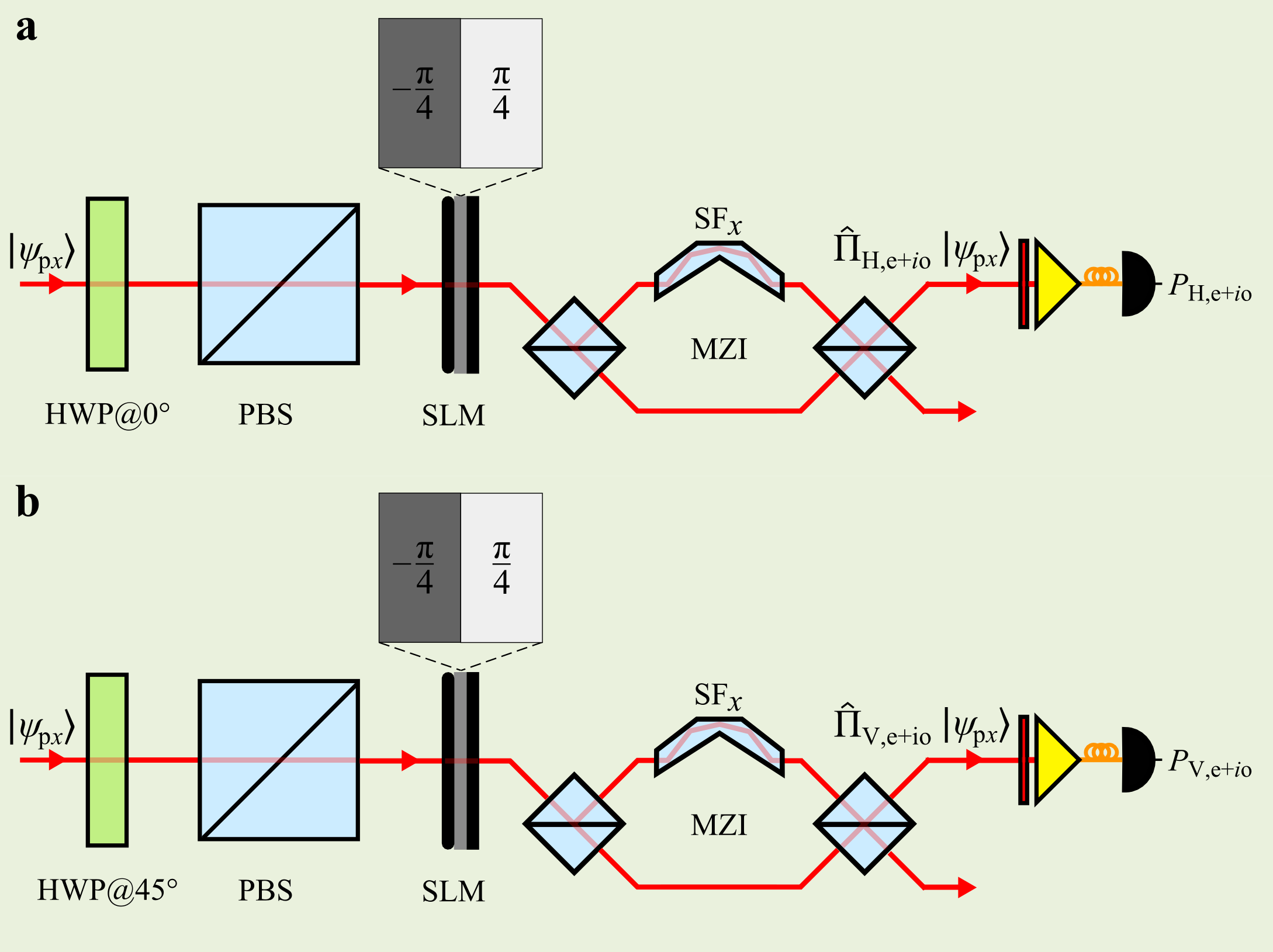


**Supplementary Figure 7 |** **Measurements required to obtain** $\boldsymbol{S}_{\boldsymbol{03}}$**.** **a**, Setup to obtain $P_{H,e+io}$. **b**, Setup to obtain $P_{V,e+io}.$

1. $S_{10}$:

Measurement of $S_{10}$ requires the same operators $\hat{\Pi}_{\mathrm{He}}$ and $\hat{\Pi}_{\mathrm{Ho}}$ shown in Supplementary Fig. 5, from which we obtain $S_{10}={2P}_{\mathrm{He}}+{2P}_{\mathrm{Ho}}-1$.

1. $S_{11}$:

Measurement of $S_{11}$ requires the same operators $\hat{\Pi}_{\mathrm{Ho}}$ and $\hat{\Pi}_{\mathrm{Ve}}$ shown in Supplementary Fig. 5, from which we obtain $S_{11}={1-2P}_{\mathrm{Ho}}-{2P}_{\mathrm{Ve}}$.

1. $S_{12}$:

Measurement of $S_{12}$ requires the projections $\hat{\Pi}_{\mathrm{He}}$ and $\hat{\Pi}_{\mathrm{Ho}}$ shown in Supplementary Fig. 5, in addition to the projections $\hat{\Pi}_{H,e+o}$ and $\hat{\Pi}_{V,e+o}$ shown in Supplementary Fig. 6. Together, these projections yield $S_{12}$: $S_{12}={2P}_{H,e+o}-2P_{V,e+o}-{2P}_{\mathrm{He}}-{2P}_{\mathrm{Ho}}+1$.

1. $S_{13}$:

Measurement of $S_{13}$ requires the projections $\hat{\Pi}_{\mathrm{He}}$ and $\hat{\Pi}_{\mathrm{Ho}}$ shown in Supplementary Fig. 5, in addition to the projections $\hat{\Pi}_{H,e+io}$ and $\hat{\Pi}_{V,e+io}$ shown in Supplementary Fig. 7. Together, these projections yield $S_{13}$: $S_{13}={2P}_{H,e+io}-2P_{V,e+io}-{2P}_{\mathrm{He}}-{2P}_{\mathrm{Ho}}+1$.

1. $S_{20}$:

Measurement of $S_{20}$ is carried out with the setup shown in Supplementary Fig. 8. The polarization projection along $|\left. D \right\rangle$ consists of a HWP with the fast axis oriented at 22.5°, followed by a PBS. The parity projection consists of an SLM imparting no phase, followed by a balanced MZI containing in one arm a spatial flipper along $x$. At the MZI output ports, the following projection operators are implemented:

$\hat{\Pi}_{\mathrm{De}}=\frac{1}{2}\left( \begin{matrix} 1 & 0 & 1 & 0 \\ 0 & 0 & 0 & 0 \\ 1 & 0 & 1 & 0 \\ 0 & 0 & 0 & 0 \end{matrix} \right)$, $\hat{\Pi}_{\mathrm{Do}}=\frac{1}{2}\left( \begin{matrix} 0 & 0 & 0 & 0 \\ 0 & 1 & 0 & 1 \\ 0 & 0 & 0 & 0 \\ 0 & 1 & 0 & 1 \end{matrix} \right)$. (7)

Measurements of $P_{\mathrm{De}}$ and $P_{\mathrm{Do}}$yield $S_{20}$: $S_{20}={2P}_{\mathrm{De}}+P_{\mathrm{Do}}-1$.


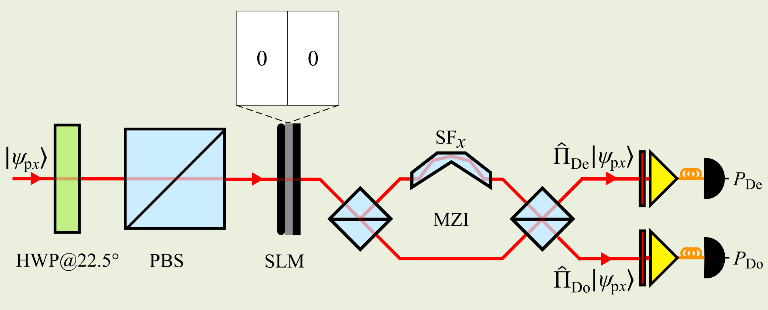


**Supplementary Figure 8 | Measurements required to obtain** $\boldsymbol{S}_{\boldsymbol{20}}$**.** Setup to obtain $P_{De}$ and $P_{Do}$.

1. $S_{21}$:

Measurement of $S_{21}$ requires the projections $\hat{\Pi}_{\mathrm{He}}$ and $\hat{\Pi}_{\mathrm{Ve}}$ shown in Supplementary Fig. 5, in addition to the projections $\hat{\Pi}_{\mathrm{De}}$ and $\hat{\Pi}_{\mathrm{Do}}$ shown in Supplementary Fig. 8. Together, these measurements yield $S_{21}$: $S_{21}={2P}_{\mathrm{De}}-2P_{\mathrm{Do}}-{2P}_{\mathrm{He}}-{2P}_{\mathrm{Ve}}+1$.

1. $S_{22}$:

Measurement of $S_{22}$ requires the projections $\hat{\Pi}_{H,e+o}$ and $\hat{\Pi}_{V,e+o}$ shown in Supplementary Fig. 6, in addition to the projections $\hat{\Pi}_{\mathrm{De}}$ and $\hat{\Pi}_{\mathrm{Do}}$ shown in Supplementary Fig. 8, and $\hat{\Pi}_{D,e+o}$ shown in Supplementary Fig. 9. The projection $\hat{\Pi}_{D,e+o}$ consists of a polarization projection along $|\left. D \right\rangle$ followed by a parity projection along $|\left. e+o \right\rangle$. The following projection operator is thus implemented:

$\hat{\Pi}_{D,e+o}=\frac{1}{4}\left( \begin{matrix} 1 & 1 & 1 & 1 \\ 1 & 1 & 1 & 1 \\ 1 & 1 & 1 & 1 \\ 1 & 1 & 1 & 1 \end{matrix} \right)$. (8)

Measurements of $P_{D,e+o}$, $P_{\mathrm{De}}$, $P_{\mathrm{Do}}$, $P_{H,e+o}$, and $P_{V,e+o}$ yield $S_{22}$: $S_{22}={4P}_{D,e+o}-2P_{\mathrm{De}}-2P_{\mathrm{Do}}-2P_{H,e+o}-2P_{V,e+o}+1$.


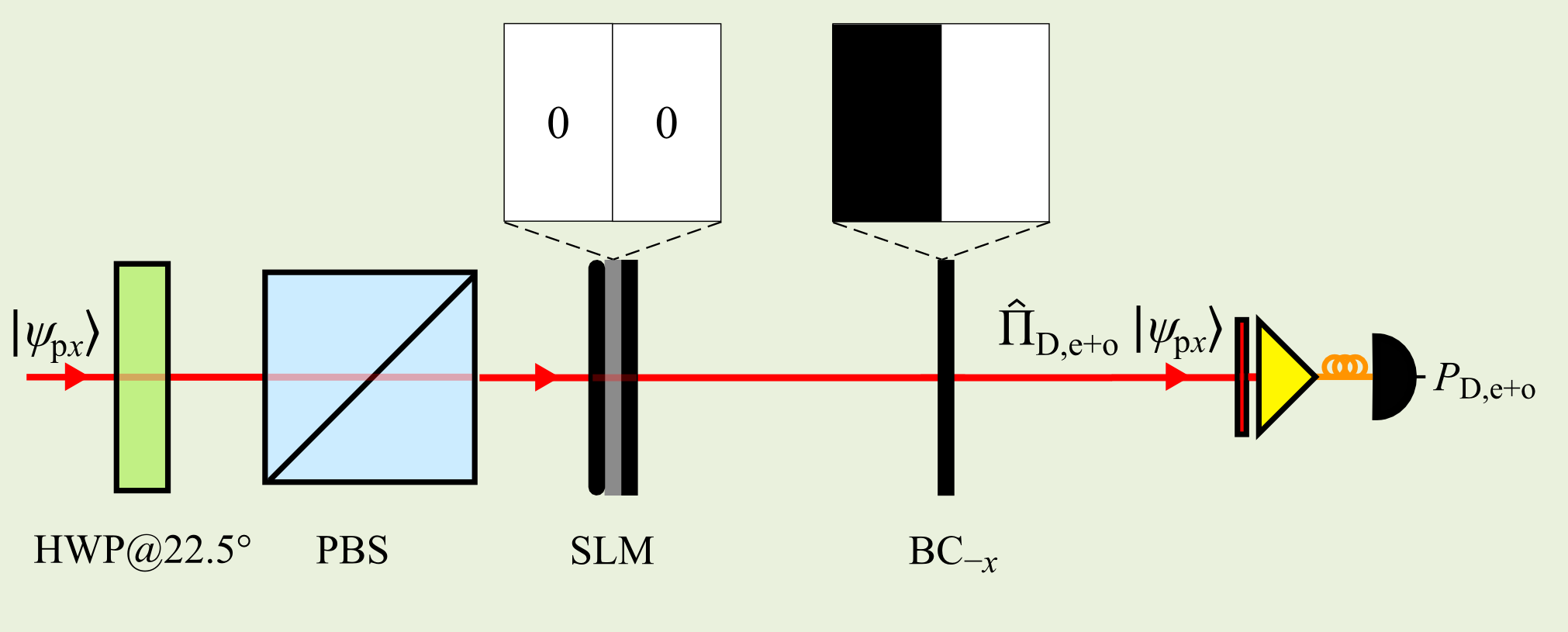


**Supplementary Figure 9 | Setup required to implement the projection operator** ${\hat{\boldsymbol{\Pi}}}_{\boldsymbol{D,e+o}}$**.**

1. $S_{23}$:

Measurement of $S_{23}$ requires the projections $\hat{\Pi}_{H,e+io}$ and $\hat{\Pi}_{V,e+io}$ shown in Supplementary Fig. 7, in addition to the projections $\hat{\Pi}_{\mathrm{De}}$ and $\hat{\Pi}_{\mathrm{Do}}$ shown in Supplementary Fig. 8, and $\hat{\Pi}_{D,e+io}$ shown in Supplementary Fig. 10. The latter projection $\hat{\Pi}_{D,e+io}$ consists of a polarization projection along $|\left. D \right\rangle$ followed by a parity projection along $|\left. e+io \right\rangle$. The following projection operator is thus implemented:

$\hat{\Pi}_{D,e+io}=\frac{1}{4}\left( \begin{matrix} 1 & -i & 1 & -i \\ i & 1 & i & 1 \\ 1 & -i & 1 & -i \\ i & 1 & i & 1 \end{matrix} \right)$. (9)

Measurements of $P_{D,e+io}$, $P_{\mathrm{De}}$, $P_{\mathrm{Do}}$, $P_{H,e+io}$, and $P_{V,e+io}$ yield $S_{23}$: $S_{23}={4P}_{D,e+io}-2P_{\mathrm{De}}-2P_{\mathrm{Do}}-2P_{H,e+io}-2P_{V,e+io}+1$.


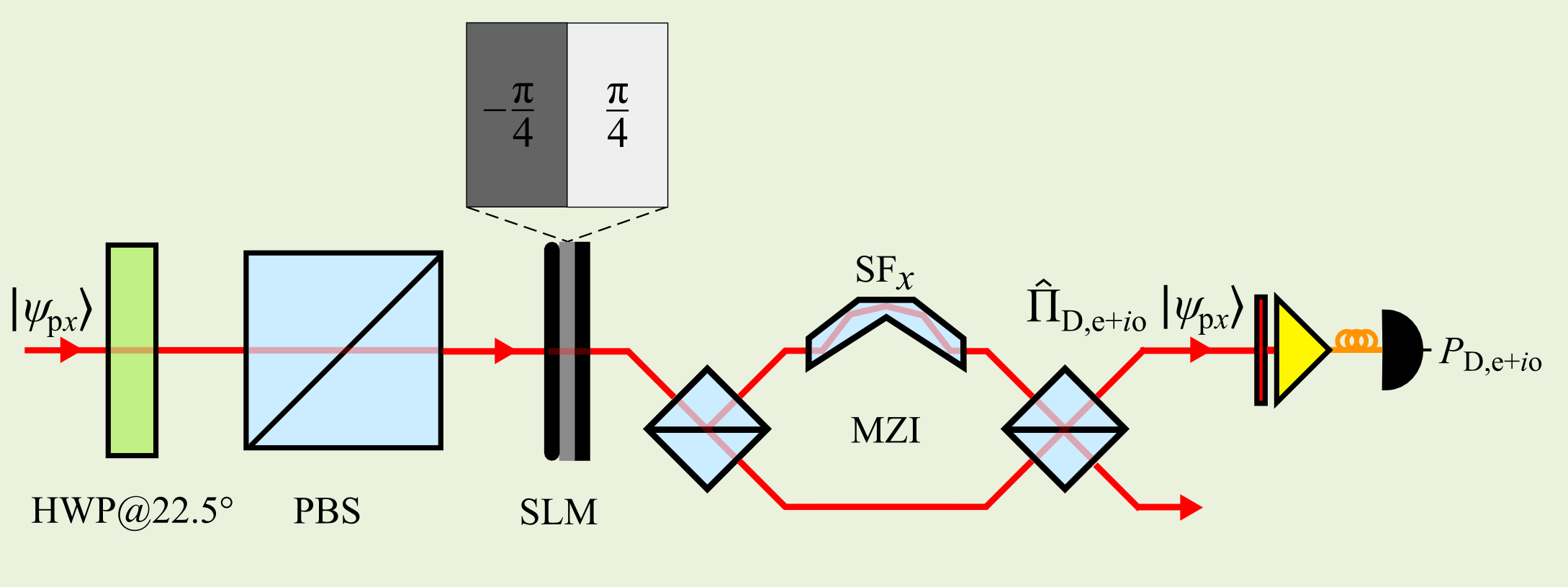


**Supplementary Figure 10 | Setup required to implement the projection operator** ${\hat{\boldsymbol{\Pi}}}_{\boldsymbol{D,e+io}}$.

1. $S_{30}$:

Measurement of $S_{30}$ is carried out utilizing the configuration shown in Supplementary Fig. 11. The polarization projection consists of a quarter-wave plate (QWP) with the fast axis oriented at 45°, followed by a PBS to project along $|\left. R \right\rangle$. The parity projection along $|\left. e \right\rangle$ and $|\left. o \right\rangle$ is shown in Supplementary Fig. 5. The following projection operators are thus implemented:

$\hat{\Pi}_{\mathrm{Re}}=\frac{1}{2}\left( \begin{matrix} 1 & 0 & -i & 0 \\ 0 & 0 & 0 & 0 \\ i & 0 & 1 & 0 \\ 0 & 0 & 0 & 0 \end{matrix} \right)$, $\hat{\Pi}_{\mathrm{Ro}}=\frac{1}{2}\left( \begin{matrix} 0 & 0 & 0 & 0 \\ 0 & 1 & 0 & -i \\ 0 & 0 & 0 & 0 \\ 0 & i & 0 & 1 \end{matrix} \right)$. (10)

Measurements of $P_{\mathrm{Re}}$ and $P_{\mathrm{Ro}}$yield $S_{30}$: $S_{30}={2P}_{\mathrm{Re}}+P_{\mathrm{Ro}}-1$.


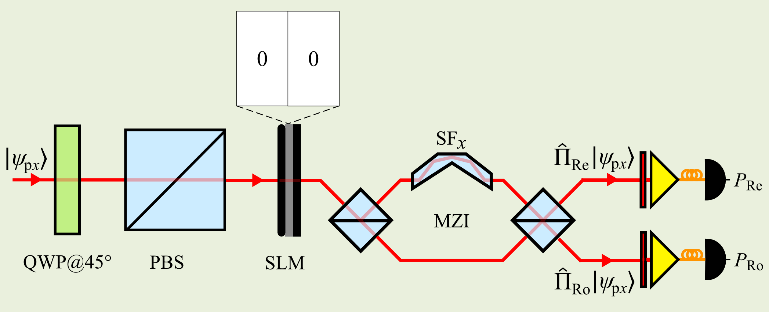


**Supplementary Figure 11 | Configuration required to measure** $\boldsymbol{S}_{\boldsymbol{30}}$**.**

1. $S_{31}$:

Measurement of $S_{31}$ requires the projections $\hat{\Pi}_{\mathrm{He}}$ and $\hat{\Pi}_{\mathrm{Ve}}$ shown in Supplementary Fig. 5, in addition to the projections $\hat{\Pi}_{\mathrm{Re}}$ and $\hat{\Pi}_{\mathrm{Ro}}$ shown in Supplementary Fig. 11. Together, these projections yield $S_{31}$: $S_{31}={2P}_{\mathrm{Re}}-2P_{\mathrm{Ro}}-{2P}_{\mathrm{He}}-{2P}_{\mathrm{Ve}}+1$.

1. $S_{32}$:

Measurement of $S_{32}$ requires the projections $\hat{\Pi}_{H,e+o}$ and $\hat{\Pi}_{V,e+o}$ shown in Supplementary Fig. 6, in addition to the projections $\hat{\Pi}_{\mathrm{Re}}$ and $\hat{\Pi}_{\mathrm{Ro}}$ shown in Supplementary Fig. 11, and $\hat{\Pi}_{R,e+o}$ shown in Supplementary Fig. 12. The projection $\hat{\Pi}_{R,e+o}$ comprises of a polarization projection along $|\left. R \right\rangle$ followed by a parity projection along $|\left. e+o \right\rangle$. The following projection operator is thus implemented:

$\hat{\Pi}_{R,e+o}=\frac{1}{4}\left( \begin{matrix} 1 & 1 & -i & -i \\ 1 & 1 & -i & -i \\ i & i & 1 & 1 \\ i & i & 1 & 1 \end{matrix} \right)$. (11)

Measurements of $P_{R,e+o}$, $P_{\mathrm{Re}}$, $P_{\mathrm{Ro}}$, $P_{H,e+o}$, and $P_{V,e+o}$ yield $S_{32}$: $S_{32}={4P}_{R,e+o}-2P_{\mathrm{Re}}-2P_{\mathrm{Ro}}-2P_{H,e+o}-2P_{V,e+o}+1$.


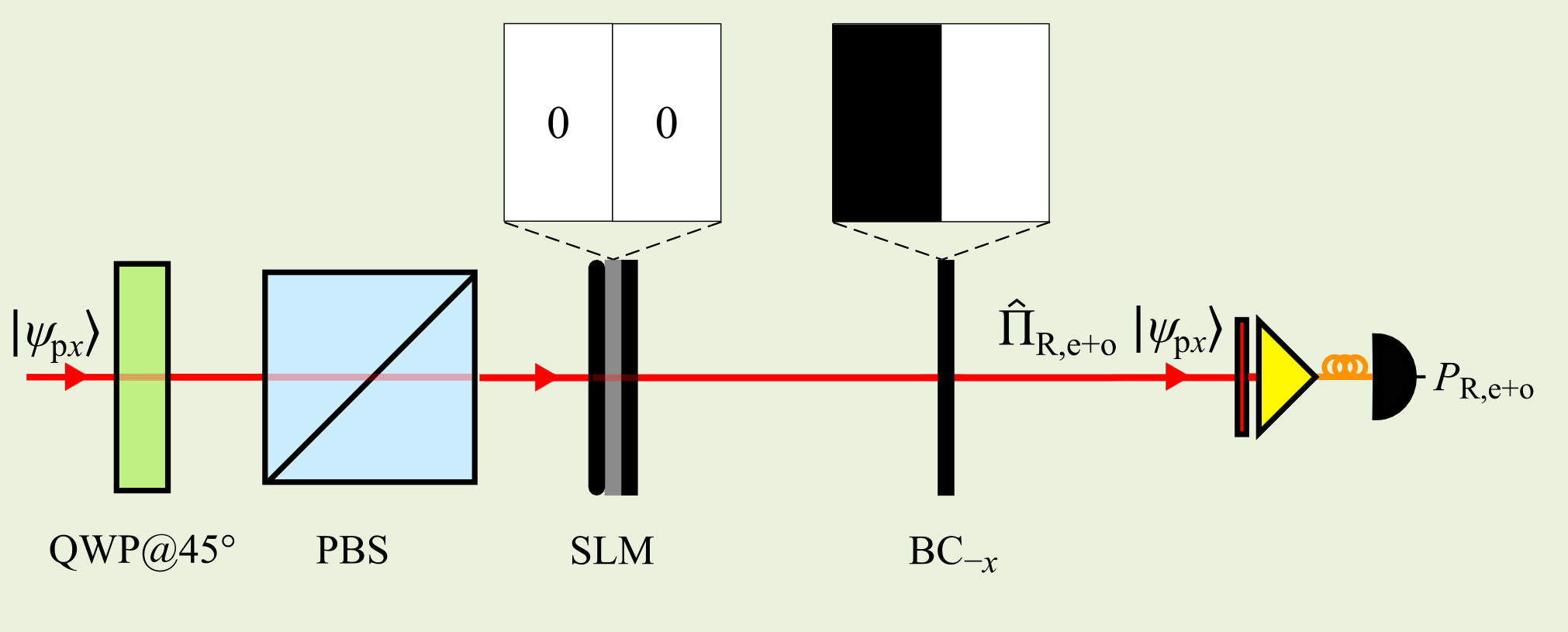


**Supplementary Figure 12 | Configuration for realizing the projection operator** ${\hat{\boldsymbol{\Pi}}}_{\boldsymbol{R,e+o}}$**.**

1. $S_{33}$:

Measurement of $S_{33}$ requires the projections $\hat{\Pi}_{H,e+io}$ and $\hat{\Pi}_{V,e+io}$ shown in Supplementary Fig. 7, in addition to the projections $\hat{\Pi}_{\mathrm{Re}}$ and $\hat{\Pi}_{\mathrm{Ro}}$ shown in Supplementary Fig. 11, and $\hat{\Pi}_{R,e+io}$ shown in Supplementary Fig. 13. The projection $\hat{\Pi}_{R,e+io}$ comprises of a polarization projection along $|\left. R \right\rangle$ followed by a parity projection along $|\left. e+io \right\rangle$. The operator implemented is thus:

$\hat{\Pi}_{R,e+io}=\frac{1}{4}\left( \begin{matrix} 1 & -i & -i & -1 \\ i & 1 & 1 & -i \\ i & 1 & 1 & -i \\ -1 & i & i & 1 \end{matrix} \right)$. (12)

Measurements of $P_{R,e+io}$, $P_{\mathrm{Re}}$, $P_{\mathrm{Ro}}$, $P_{H,e+io}$, and $P_{V,e+io}$ yield $S_{33}$: $S_{33}={4P}_{R,e+io}-2P_{\mathrm{Re}}-2P_{\mathrm{Ro}}-2P_{H,e+io}-2P_{V,e+io}+1$.


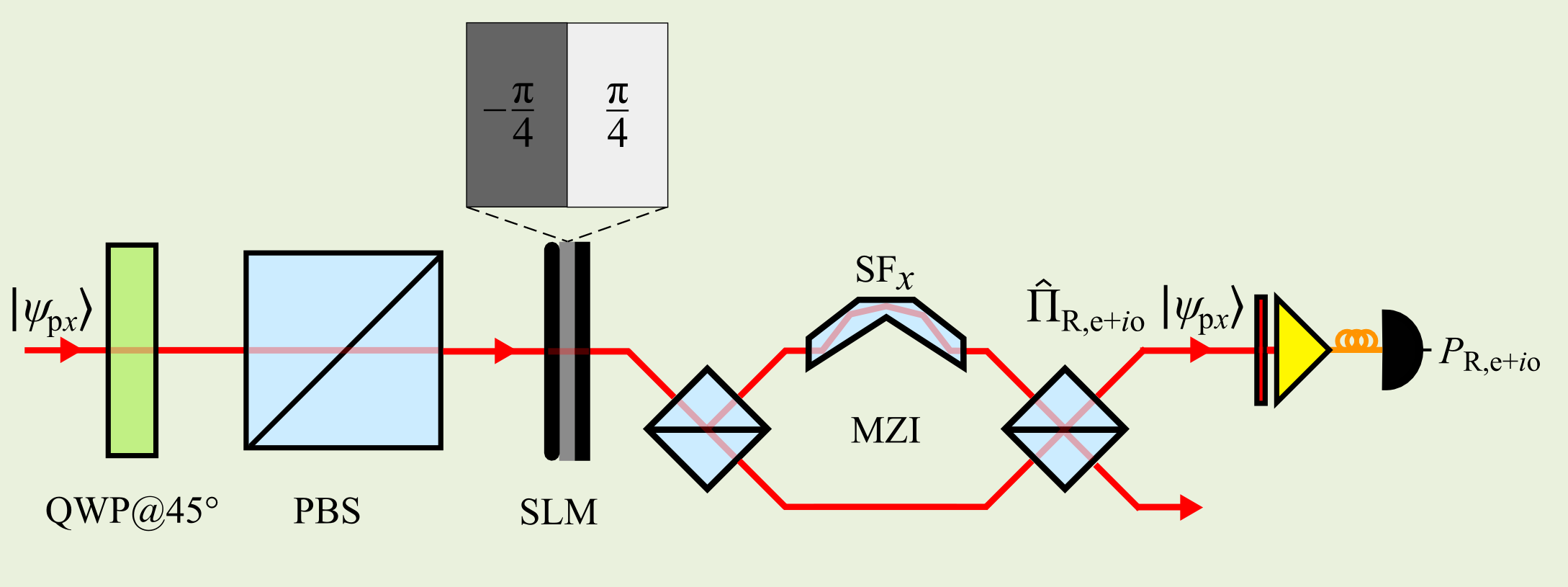


**Supplementary Figure 13 | Configuration for measuring the projection operator** ${\hat{\boldsymbol{\Pi}}}_{\boldsymbol{R(e+io)}}$**.**

**Supplementary Note 4 | Three-qubit projections for quantum state tomography in the joint Hilbert space of polarization, *x*-parity, and *y*-parity**

We express a three-qubit density matrix $\hat{\rho}$ as follows:

$\hat{\rho}=\frac{1}{8}\sum_{j,k,l=0}^{3} S_{jkl}\hat{\sigma}_{j}\bigotimes\hat{\sigma}_{k}\bigotimes\hat{\sigma}_{l}$, (13)

where $\left\{ \hat{\sigma}_{j} \right\}$ are the usual Pauli matrices on the subspaces for each of the three qubits,

$\hat{\sigma}_{0}=\left( \begin{matrix} 1 & 0 \\ 0 & 1 \end{matrix} \right)$, $\hat{\sigma}_{1}=\left( \begin{matrix} 0 & 1 \\ 1 & 0 \end{matrix} \right)$, $\hat{\sigma}_{2}=\left( \begin{matrix} 0 & i \\ -i & 0 \end{matrix} \right)$, $\hat{\sigma}_{3}=\left( \begin{matrix} 1 & 0 \\ 0 & -1 \end{matrix} \right)$, (14)

and $\left\{ S_{jkl} \right\}$ can be considered three-qubit ‘Stokes parameters’, an extension to three qubits of the two-qubit Stokes parameters previously studied in Refs. [3-5]. The values for the three-qubit Stokes parameters $S_{jkl}$ are determined through

$S_{jkl}=\mathrm{Tr}\left\{ \left( \hat{\sigma}_{j}\bigotimes\hat{\sigma}_{k}\bigotimes\hat{\sigma}_{l} \right)\hat{\rho} \right\}$, (15)

which can be obtained experimentally via projective measurements carried out in cascade over the spaces associated with each of the three qubits. In our case of a three-qubit state comprising of polarization, *x*-parity, and *y*-parity qubits, such a scheme would take the general form shown in Supplementary Fig. 14. Obtaining $\left\{ S_{jkl} \right\}$ allows us to reconstruct $\hat{\rho}$.


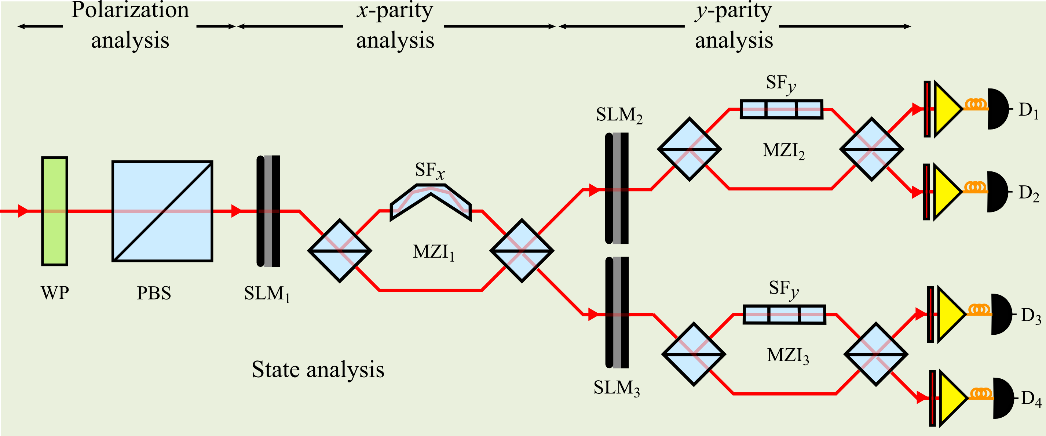


**Supplementary Figure 14 | Three-qubit projective measurements via a cascade of one-qubit projections.** From left to right, projections are carried out on the polarization, $x$-parity, and $y$-parity spaces. WP: wave-plate; PBS: polarizing beam splitter; SLM: spatial light modulator; SF: spatial flipper implemented by a parity prism, operating either on $x$-parity (SF_x_) or $y$-parity (SF_y_); MZI: Mach-Zehnder interferometer; D_1_ through D_4_: single-photon-sensitive detectors. SLM_1_ through SLM_3_ are not polarization sensitive. At the output ports, light is passed through a spectral filter, and then coupled into fibers that deliver the photons to the detectors.

This direct scheme poses considerable challenges, such as the precise alignment of multiple interferometers. We thus use an alternate scheme that makes use of only one interferometer, whose configuration is adjusted for the different projections via appropriate modification of the spatial flipper orientation. Only the spatial-parity projections require this approach, while polarization analysis is carried out in the usual manner, by making projections along the $\left\{ |\left. H \right\rangle,|\left. V \right\rangle\right\}$, $\left\{ |\left. H\pm V \right\rangle\right\}$, and $\left\{ |\left. H\pm iV \right\rangle\right\}$ bases.

Here, we outline our approach for measuring the 16 two-qubit Stokes parameters $\left\{ S_{kl} \right\}$ required for the analysis of *x*- and *y*-parity qubits (with the polarization index held fixed). These measurements are to be combined with the polarization projections to obtain the full set of 64 three-qubit Stokes parameters.

1. $S_{00}$:

Measurement of $S_{00}$ is carried out with the setup shown in Supplementary Fig. 15. An SLM imparting no phase is followed by a balanced Mach-Zehnder interferometer (MZI) containing in one arm a spatial flipper along $x$. At the two output ports, the following projection operators are implemented:

$\hat{\Pi}_{1}=\left( \begin{matrix} 1 & 0 & 0 & 0 \\ 0 & 1 & 0 & 0 \\ 0 & 0 & 0 & 0 \\ 0 & 0 & 0 & 0 \end{matrix} \right)$, $\hat{\Pi}_{2}=\left( \begin{matrix} 0 & 0 & 0 & 0 \\ 0 & 0 & 0 & 0 \\ 0 & 0 & 1 & 0 \\ 0 & 0 & 0 & 1 \end{matrix} \right)$. (16)

Measurements $P_{1}$ and $P_{2}$ at the outputs yield $S_{00}$: $S_{00}=P_{1}+P_{2}$.


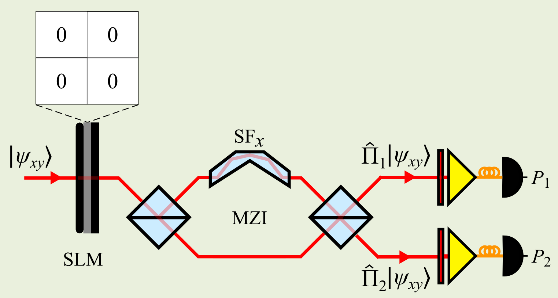


**Supplementary Figure 15 | Measurement of** $\boldsymbol{S}_{\boldsymbol{00}}$**.**

1. $S_{01}$:

Measurement of $S_{01}$ is carried out with the setup shown in Supplementary Fig. 16. The SLM imparts no phase, but a beam clipper blocks light in lower half of the plane along the $y$ (BC_-_*_y_*), such that the operator implemented is:

$\hat{\Pi}_{3}=\left( \begin{matrix} 1 & 1 & 0 & 0 \\ 1 & 1 & 0 & 0 \\ 0 & 0 & 1 & 1 \\ 0 & 0 & 1 & 1 \end{matrix} \right)$, (17)

and we have $S_{01}=2P_{3}-1$.


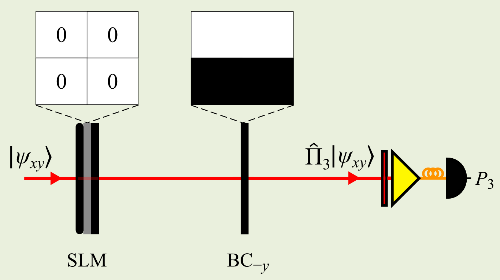


**Supplementary Figure 16 | Measurement of** $\boldsymbol{S}_{\boldsymbol{01}}$**.**

1. $S_{02}$:

Measurement of $S_{02}$ is carried out with the setup shown in Supplementary Fig. 17. An SLM implements a $\frac{\pi}{2}$ phase step along $y$ to implement a $\frac{\pi}{2}$ rotation for the $y$-parity qubit, followed by a MZI with a spatial flipper along $y$. The operator implemented is:

$\hat{\Pi}_{4}=\left( \begin{matrix} 1 & i & 0 & 0 \\ -i & 1 & 0 & 0 \\ 0 & 0 & 1 & i \\ 0 & 0 & -i & 1 \end{matrix} \right)$, (18)

and we have $S_{02}=2P_{4}-1$.


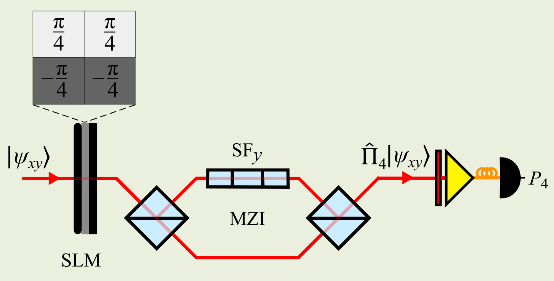


**Supplementary Figure 17 | Measurement of** $\boldsymbol{S}_{\boldsymbol{02}}$**.**

1. $S_{03}$:

Measurement of $S_{03}$ is carried out with the setup shown in Supplementary Fig. 18. The SLM imparts no phase, followed by a MZI with a spatial flipper along $y$ in one arm. The operator implemented is:

$\hat{\Pi}_{5}=\left( \begin{matrix} 1 & 0 & 0 & 0 \\ 0 & 0 & 0 & 0 \\ 0 & 0 & 1 & 0 \\ 0 & 0 & 0 & 0 \end{matrix} \right)$, (19)

and we have $S_{03}=2P_{5}-1$.


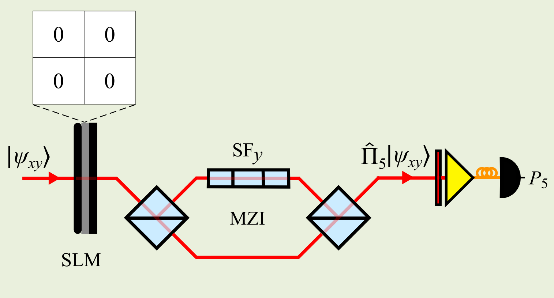


**Supplementary Figure 18 | Measurement of** $\boldsymbol{S}_{\boldsymbol{03}}$**.**

1. $S_{10}$:

Measurement of $S_{10}$ is carried out with the setup shown in Supplementary Fig. 19. The SLM imparts no phase, followed by a beam clipper blocking the $-x$ plane half (BC_-_*_x_*). The operator implemented is thus:

$\hat{\Pi}_{6}=\left( \begin{matrix} 1 & 0 & 1 & 0 \\ 0 & 1 & 0 & 1 \\ 1 & 0 & 1 & 0 \\ 0 & 1 & 0 & 1 \end{matrix} \right)$, (20)

and we have $S_{10}=2P_{6}-1$.


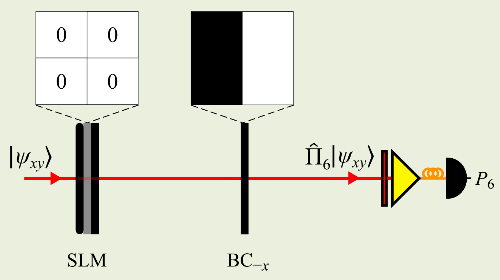


**Supplementary Figure 19 | Measurement of** $\boldsymbol{S}_{\boldsymbol{10}}$**.**

1. $S_{11}$:

Measurement of $S_{11}$ is carried out with the setups shown in Supplementary Fig. 20. First, an SLM imparting no phase is followed by a beam clipper blocking the $-x$ and $-y$ plane halves (BC_-_*_x,-y_*). an SLM imparting no phase is followed by a beam clipper blocking the $+x$ and $+y$ plane halves (BC_+_*_x,+y_*). The operators implemented are:

$\hat{\Pi}_{7}=\left( \begin{matrix} 1 & 1 & 1 & 1 \\ 1 & 1 & 1 & 1 \\ 1 & 1 & 1 & 1 \\ 1 & 1 & 1 & 1 \end{matrix} \right)$, $\hat{\Pi}_{8}=\left( \begin{matrix} 1 & -1 & -1 & 1 \\ -1 & 1 & 1 & -1 \\ -1 & 1 & 1 & -1 \\ 1 & -1 & -1 & 1 \end{matrix} \right)$, (21)

and we have $S_{11}=2P_{7}+2P_{8}-1$.


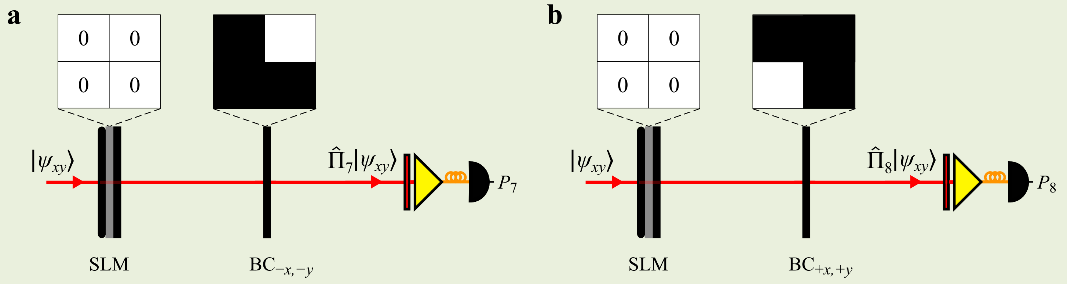


**Supplementary Figure 20 | Measurements required to obtain** $\boldsymbol{S}_{\boldsymbol{11}}$**.**

1. $S_{12}$:

Measurement of $S_{12}$ is carried out with the setup shown in Supplementary Fig. 21. An SLM provides a $\frac{\pi}{2}$-phase step along $x$ and $y$, thereby implementing a joint rotation of $\frac{\pi}{2}$ for both the *x*-parity and *y*-parity qubits, is followed by a MZI with a spatial flipper along $y$. The operator implemented is:

$\hat{\Pi}_{9}=\left( \begin{matrix} 1 & 0 & 0 & i \\ 0 & 1 & -i & 1 \\ 1 & i & 1 & 0 \\ -i & 0 & 0 & 1 \end{matrix} \right)$, (22)

and we have $S_{12}=2P_{9}-1$.


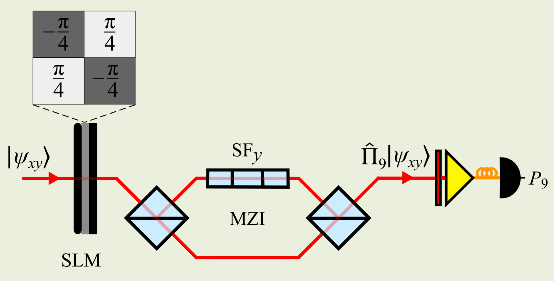


**Supplementary Figure 21 | Measurement of** $\boldsymbol{S}_{\boldsymbol{12}}$**.**

1. $S_{13}$:

Measurement of $S_{13}$ is carried out with the setups shown in Supplementary Fig. 22. An SLM imparts no phase is followed by a MZI with a spatial flipper along $y$. Beam clippers that block the $-x$ and $+x$ plane halves (BC_-_*_x_* and BC_+_*_x_*, respectively) are placed at the outputs of the MZI. The operators implemented are:

$\hat{\Pi}_{10}=\left( \begin{matrix} 1 & 0 & 1 & 0 \\ 0 & 0 & 0 & 0 \\ 1 & 0 & 1 & 0 \\ 0 & 0 & 0 & 0 \end{matrix} \right)$, $\hat{\Pi}_{11}=\left( \begin{matrix} 0 & 0 & 0 & 0 \\ 0 & 1 & 0 & -1 \\ 0 & 0 & 0 & 0 \\ 0 & -1 & 0 & 1 \end{matrix} \right)$, (23)

and we have $S_{13}=2P_{10}+2P_{11}-1$.


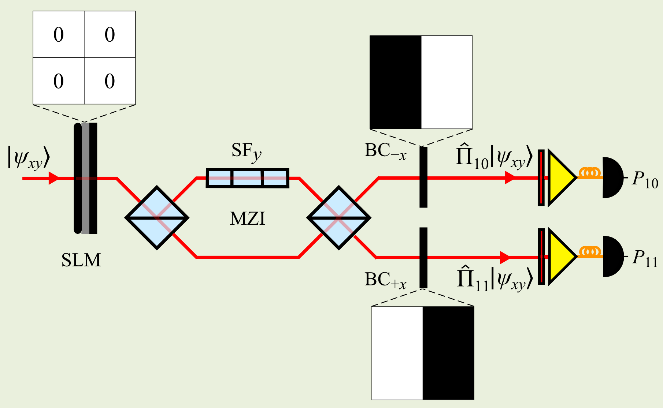


**Supplementary Figure 22 | Measurements required to obtain** $\boldsymbol{S}_{\boldsymbol{13}}$**.**

1. $S_{20}$:

Measurement of $S_{20}$ is carried out with the setup shown in Supplementary Fig. 23. An SLM imparts a $\frac{\pi}{2}$-phase step along $x$ and none along $y$, hence implementing a $\frac{\pi}{2}$-rotation for the $x$-parity qubit, is followed by a MZI with a spatial flipper along $x$. The operator implemented is:

$\hat{\Pi}_{12}=\left( \begin{matrix} 1 & 0 & i & 0 \\ 0 & 1 & 0 & i \\ -i & 0 & 1 & 0 \\ 0 & -i & 0 & 1 \end{matrix} \right)$, (24)

and we have $S_{20}=2P_{12}-1$.


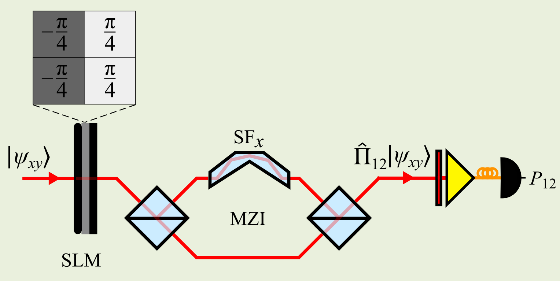


**Supplementary Figure 23 | Measurements required to obtain** $\boldsymbol{S}_{\boldsymbol{20}}$**.**

1. $S_{21}$:

Measurement of $S_{21}$ is carried out with the setup shown in Supplementary Fig. 24. An SLM imparts a $\frac{\pi}{2}$-phase step along $x$ and $y$, hence implementing a $\frac{\pi}{2}$-rotation for the joint $x$- and $y$-parity qubits, is followed by a MZI with a spatial flipper along $x$. The operator implemented is:

$\hat{\Pi}_{13}=\left( \begin{matrix} 1 & 0 & 0 & i \\ 0 & 1 & i & 0 \\ 0 & -i & 1 & 0 \\ -i & 0 & 0 & 1 \end{matrix} \right)$, (25)

and we have $S_{21}=2P_{13}-1$.


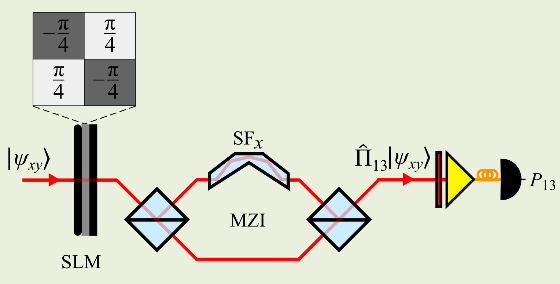


**Supplementary Figure 24 | Measurements required to obtain** $\boldsymbol{S}_{\boldsymbol{21}}$**.**

1. $S_{22}$:

Measurement of $S_{22}$ is carried out with the setup shown in Supplementary Fig. 25. An SLM imparting a $\frac{\pi}{2}$-phase step along $x$ and $y$ separately, hence implementing a separable $\frac{\pi}{2}$-rotation for each of the $x$-parity and $y$-parity qubits, is followed by a MZI containing a spatial flipper along both $x$ and $y$ in its two arms. The operator implemented is:

$\hat{\Pi}_{14}=\left( \begin{matrix} 1 & 0 & 0 & -1 \\ 0 & 1 & 1 & 0 \\ 0 & 1 & 1 & 0 \\ -1 & 0 & 0 & 1 \end{matrix} \right)$, (26)

and we have $S_{22}=2P_{14}-1$.


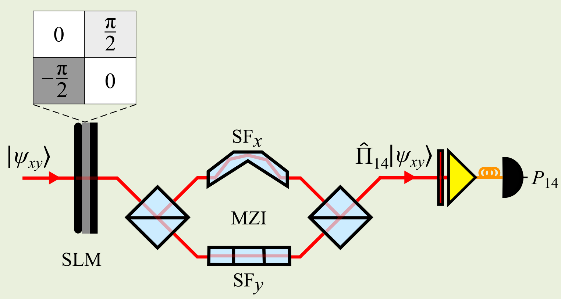


**Supplementary Figure 25 | Measurements required to obtain** $\boldsymbol{S}_{\boldsymbol{22}}$**.**

1. $S_{23}$:

Measurement of $S_{23}$ is carried out with the setup shown in Supplementary Fig. 26. An SLM imparts a $\frac{\pi}{2}$-phase step along $x$ and none along $y$, hence implementing a $\frac{\pi}{2}$-rotation for the $x$-parity qubit, is followed by a MZI containing spatial flippers along $x$ and $y$ in its two arms. The operator implemented is:

$\hat{\Pi}_{15}=\left( \begin{matrix} 1 & 0 & i & 0 \\ 0 & 1 & 0 & -i \\ -i & 0 & 1 & 0 \\ 0 & i & 0 & 1 \end{matrix} \right)$, (27)

and we have $S_{23}=2P_{15}-1$.


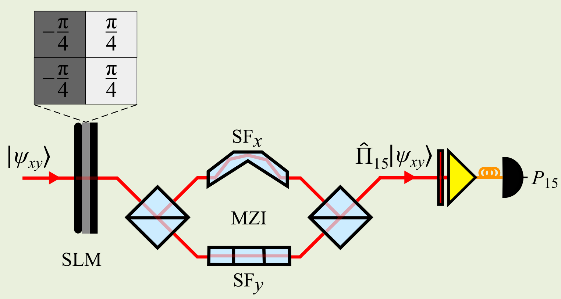


**Supplementary Figure 26 | Measurements required to obtain** $\boldsymbol{S}_{\boldsymbol{23}}$**.**

1. $S_{30}$:

Measurement of $S_{30}$ requires the same operators $\hat{\Pi}_{1}$ and $\hat{\Pi}_{2}$ shown in Supplementary Fig. 15, and $S_{30}=P_{1}-P_{2}$.

1. $S_{31}$:

Measurement of $S_{31}$ is carried out with the setup shown in Supplementary Fig. 27. An SLM imparting no phase is followed by a MZI with a spatial flipper along $x$. Beam clippers that block the $-y$ and $+y$ plane halves (BC*_-y_* and BC*_+y_*, respectively) are placed at the outputs of the MZI. The operators implemented are:

$\hat{\Pi}_{16}=\left( \begin{matrix} 1 & 1 & 0 & 0 \\ 1 & 1 & 0 & 0 \\ 0 & 0 & 0 & 0 \\ 0 & 0 & 0 & 0 \end{matrix} \right)$, $\hat{\Pi}_{17}=\left( \begin{matrix} 0 & 0 & 0 & 0 \\ 0 & 0 & 0 & 0 \\ 0 & 0 & 1 & -1 \\ 0 & 0 & -1 & 1 \end{matrix} \right)$, (28)

and we have $S_{31}=2P_{16}+2P_{17}-1$.


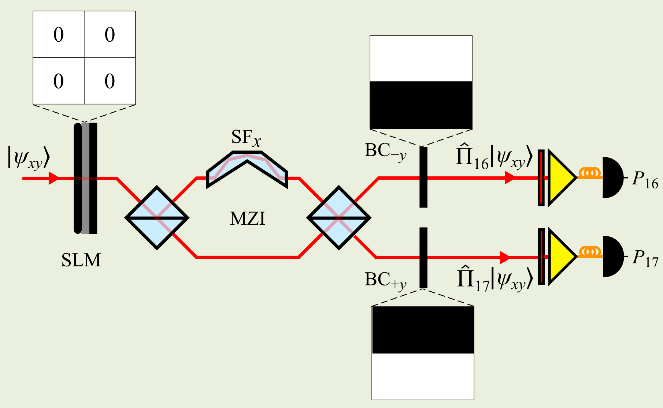


**Supplementary Figure 27 | Measurements required to obtain** $\boldsymbol{S}_{\boldsymbol{31}}$**.**

1. $S_{32}$:

Measurement of $S_{32}$ is carried out with the setup shown in Supplementary Fig. 28. An SLM imparts a $\frac{\pi}{2}$-phase step along $y$ and none along $x$, hence implementing a $\frac{\pi}{2}$-rotation for the $y$-parity qubit, is followed by a MZI with a spatial flipper along both $x$ and $y$. The operator implemented is:

$\hat{\Pi}_{18}=\left( \begin{matrix} 1 & i & 0 & 0 \\ -i & 1 & 0 & 0 \\ 0 & 0 & 1 & -i \\ 0 & 0 & i & 1 \end{matrix} \right)$, (29)

and we have $S_{32}=2P_{18}-1$.


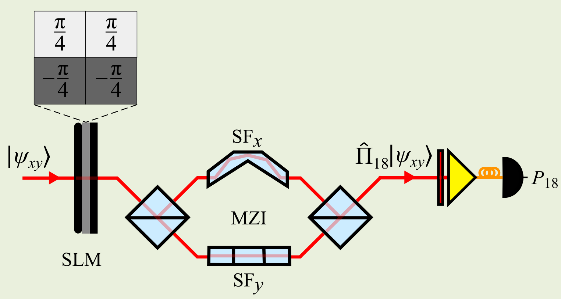


**Supplementary Figure 28 | Measurements required to obtain** $\boldsymbol{S}_{\boldsymbol{32}}$**.**

1. $S_{33}$:

Measurement of $S_{33}$ is carried out with the setup shown in Supplementary Fig. 29. An SLM imparting no phase is followed by a MZI with a spatial flipper in both $x$ and $y$. The operator implemented is:

$\hat{\Pi}_{19}=\left( \begin{matrix} 1 & 0 & 0 & 0 \\ 0 & 0 & 0 & 0 \\ 0 & 0 & 0 & 0 \\ 0 & 0 & 0 & 1 \end{matrix} \right)$, (30)

and we have $S_{33}=2P_{19}-1$.


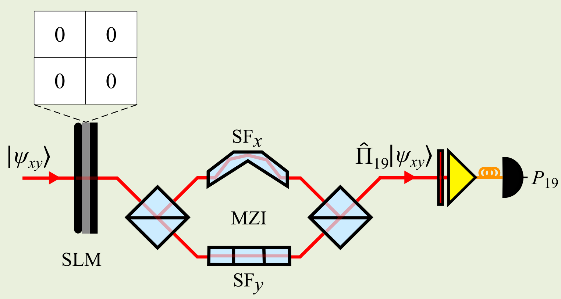


**Supplementary Figure 29 | Measurements required to obtain** $\boldsymbol{S}_{\boldsymbol{33}}$**.**

**Supplementary Methods | Details for data acquisition and coincidence measurements**

**
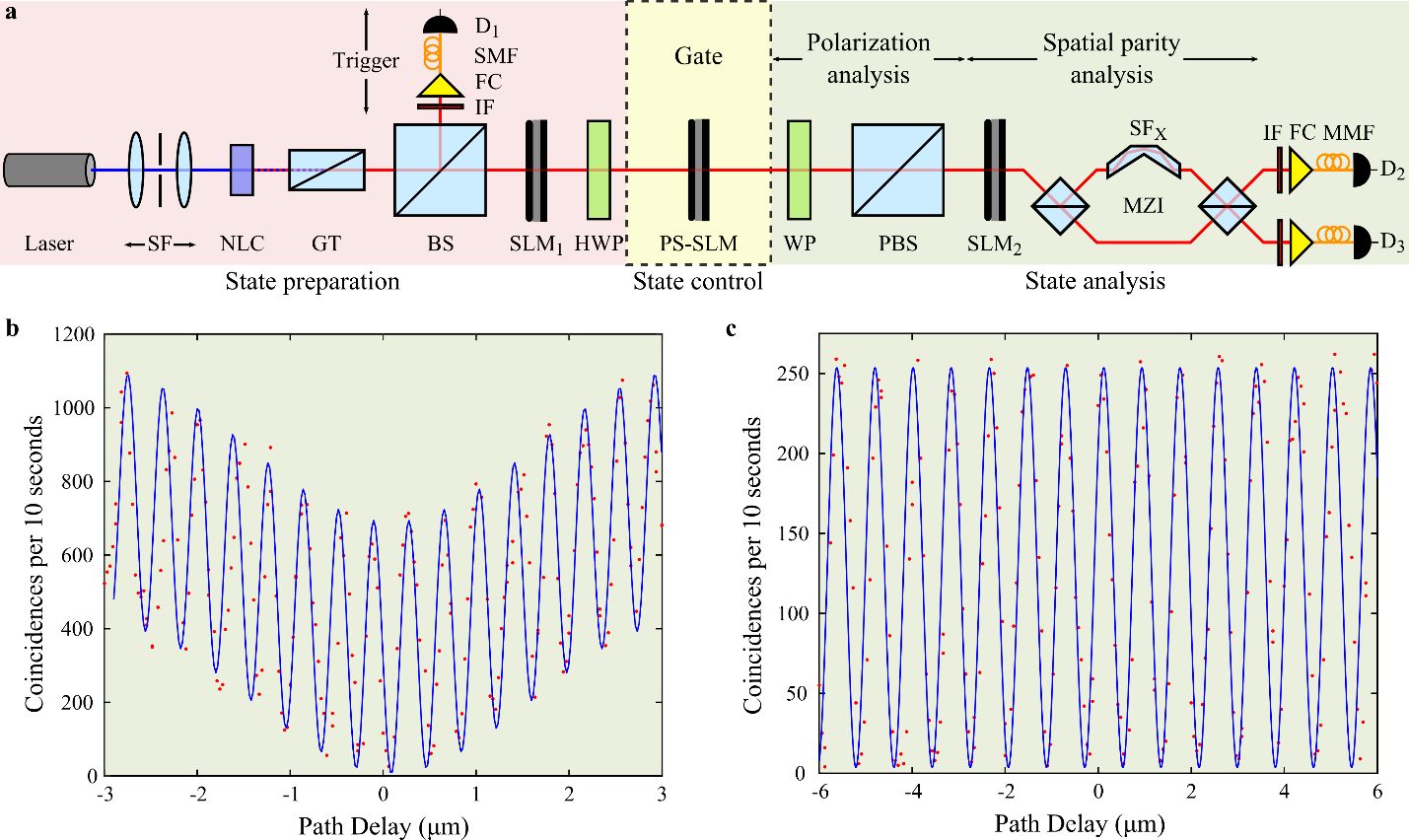
**

**Supplementary Figure 30 |** **Experimental setup highlighting the stages of state preparation, state control via a quantum gate, and state analysis.** **a,** SF: Spatial filter; NLC: nonlinear crystal; GT: Glan-Thomson polarizer; BS: beamsplitter; SLM: spatial light modulator; HWP: half-wave plate; PS-SLM: polarization-sensitive spatial light modulator; WP: wave plate (either half-wave or quarter-wave according to the measurement basis); PBS: polarizing beamsplitter; SF_X_: spatial flipper along *x*; MZI: Mach-Zehnder interferometer; IF: interference filter; FC: fiber coupler; MMF: multi-mode fiber; SMF: single-mode fiber; D_1_, D_2_, and D_3_: single-photon-sensitive detectors. The spatial flipper – implemented by the parity prism – in the MZI is varied according to the measurement basis. All measurements are carried out by recording the detection coincidences between D_1_ and D_2_. **b, c,** Two-photon, and single-photon interference, measured in coincidence, showing the quality of alignment for the MZI employed in the state analysis stage. The bi-photons in (b) are spectrally filtered with a 40 nm filter, and collected with MMFs for 10 s per point at D_2_ and D_3_, resulting in an interferogram with a visibility of 96.5%. The bi-photons in (c) are spectrally filtered with a 10 nm filter, and collected with a SMF at D_1_ and a MMF at D_2_, for 10 s per point, in a heralded configuration, resulting in an interferogram with a visibility of 97.1%.


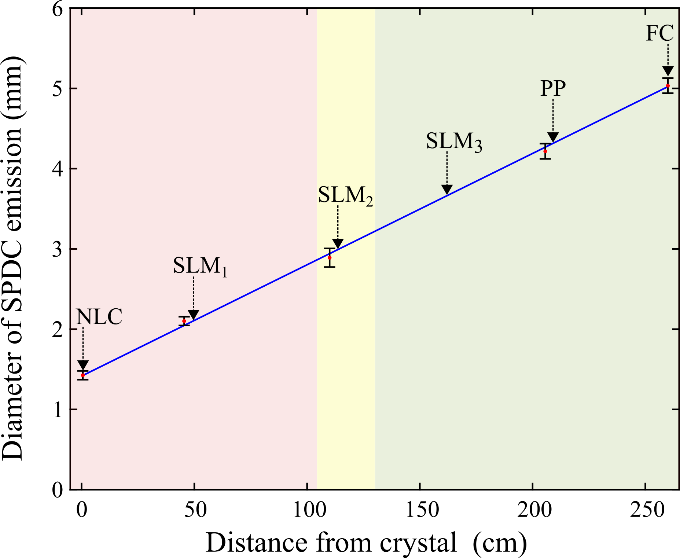


**Supplementary Figure 31 |** **Measurement of beam diameter of down-conversion with a knife-edge test at different locations along the length of the setup.** The location of certain components is indicated with an arrow. NLC: nonlinear crystal; SLM: spatial light modulator; PP: parity prism, FC: fiber coupler at the output of the MZI; MZI: Mach-Zehnder interferometer. Error bars are s.d.


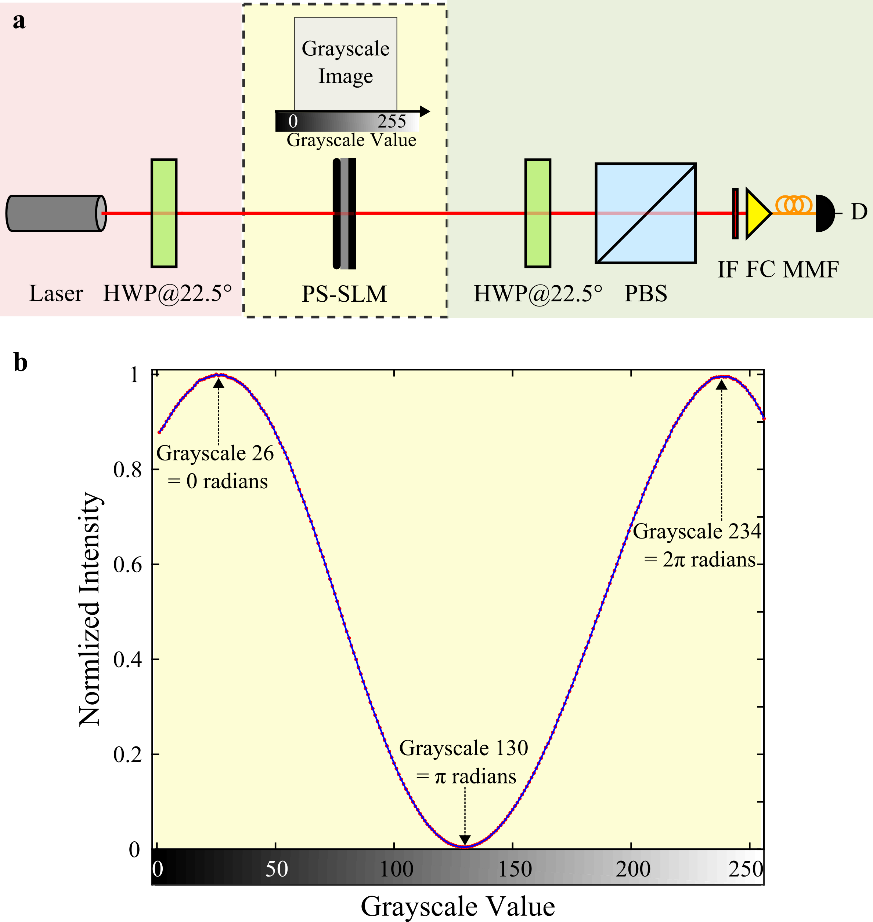


**Supplementary Figure 32 |** **Calibration of phase shift introduced by the spatial light modulator as a function of the grayscale image displayed on it.** **a,** HWP: half-wave plate; PS-SLM: polarization-sensitive spatial light modulator; PBS: polarizing beam splitter; IF: interference filter; FC: fiber coupler; MMF: multi-mode fiber; D: photodetector. An intensity modulator is implemented when diagonally polarized light reflects from the PS-SLM and is analyzed in the diagonal basis. The grayscale value displayed on the SLM ranges from 0, representing a pure black image, to 255, representing a pure white image, with varying shades of gray in between. **b,** When the phase shift between the horizontal and vertical components introduced by the SLM is zero (grayscale 26), the beam is diagonally polarized, and the intensity detected by D is maximum. As the grayscale value is flipped, the beam becomes elliptically polarized, and eventually changes its handedness. When the phase shift between the horizontal and vertical components becomes π radians (grayscale 130), the beam is anti-diagonally polarized, and the intensity detected by D is minimum. When the phase shift between the horizontal and vertical components becomes 2π radians (grayscale 234), the beam becomes diagonally polarized again, and the intensity detected by D reaches the maximum.


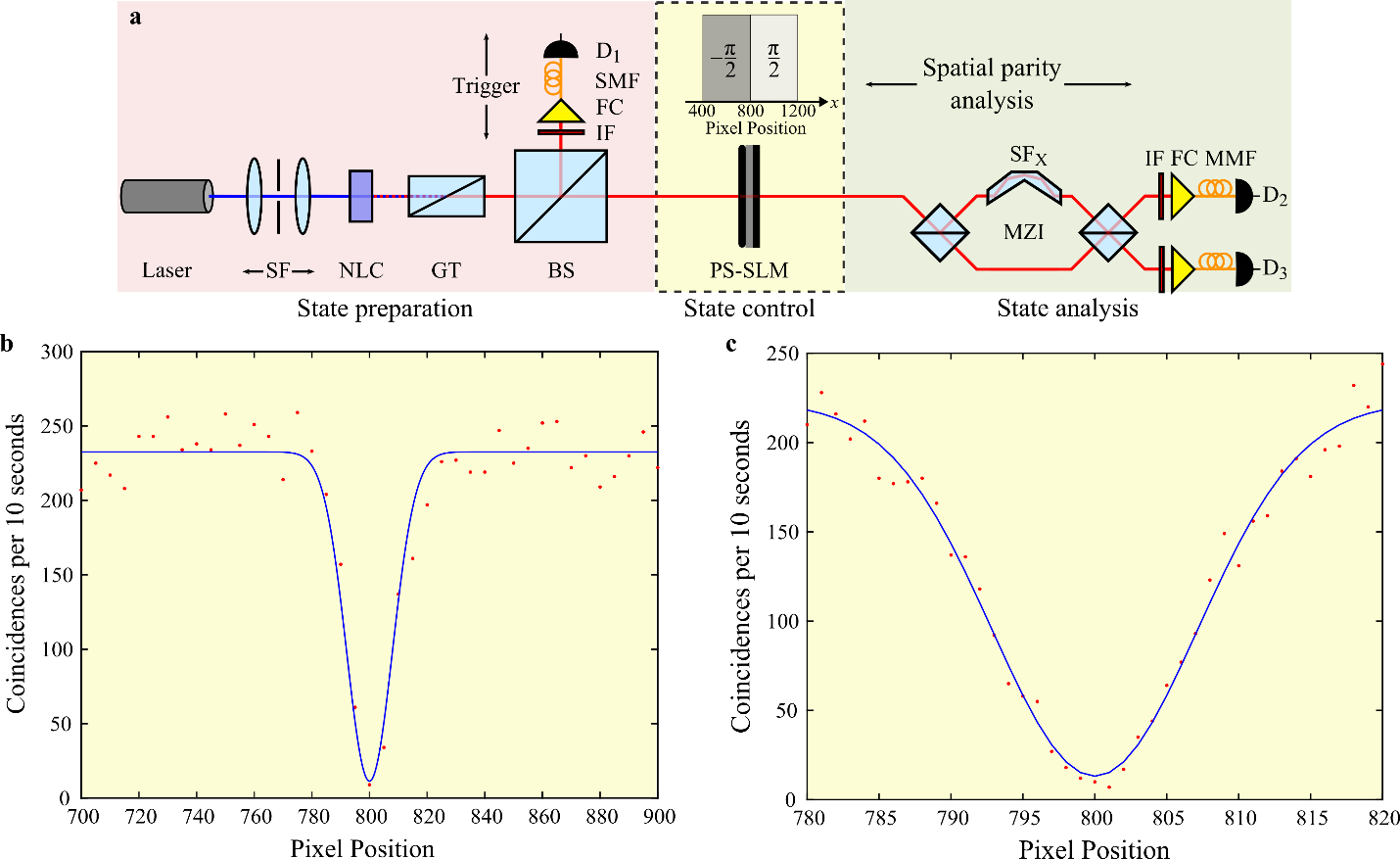


**Supplementary Figure 33 |** **Protocol for optimum positioning of phase images on the spatial light modulator**. **a,** SF: Spatial filter; NLC: nonlinear crystal; GT: Glan-Thomson polarizer; BS: beam splitter; PS-SLM: polarization-sensitive spatial light modulator; SF_X_: spatial flipper along *x*; MZI: Mach-Zehnder interferometer; IF: interference filter; FC: fiber coupler; MMF: multi-mode fiber; SMF: single-mode fiber; D_1_, D_2_, and D_3_: single-photon-sensitive detectors. The delay in the MZI is held constant for constructive interference, corresponding to projecting onto $|\left. e \right\rangle$. A phase step of π is then scanned across the SLM resulting in switching of the projection from $|\left. e \right\rangle$ to $|\left. o \right\rangle$. The pixel pitch of the SLM is 20 µm. **b, c,** Measurements with pixel step size of 5 and 1, respectively, showing that the phase image on the SLM is well centered at pixel position 800.

**Supplementary References**

[1] Padgett, M. J., and Lesso J. P. Dove prisms and polarized light. *J. Mod. Opt.*  **46.2**, 175-179 (1999).

[2] Moreno I., Gonzalo P., and Marija S. Polarization transforming properties of Dove prisms. *Opt. Commun.* **220.4**, 257-268 (2003).

[3] Abouraddy, A. F., Sergienko, A. V., Saleh, B. E. A. & Teich, M. C. Quantum entanglement and the two-photon Stokes parameters. *Opt. Commun*. **210**, 93-98 (2002).

[4] Abouraddy, A. F., Kagalwala, K. H. & Saleh, B. E. A. Two-point optical coherency matrix tomography. *Opt. Lett.* **39**, 2411-2414 (2014).

[5] Kagalwala, K. H., Kondakci, H. E., Abouraddy A. F. & Saleh, B. E. A. Optical coherency matrix tomography. *Sci. Rep.* **5**, 15333 (2015).
